# C-5 Hydroxyethyl and Hydroxypropyl Acyclonucleosides as Substrates for Thymidine Kinase of Herpes Simplex Virus Type 1 (HSV-1 TK): Syntheses and Biological Evaluation

**DOI:** 10.3390/molecules18055104

**Published:** 2013-05-02

**Authors:** Andrijana Meščić, Svjetlana Krištafor, Ivana Novaković, Amar Osmanović, Ursina Müller, Davorka Završnik, Simon M. Ametamey, Leonardo Scapozza, Silvana Raić-Malić

**Affiliations:** 1 Department of Organic Chemistry, Faculty of Chemical Engineering and Technology, University of Zagreb, Marulićev trg 20, HR-10000 Zagreb, Croatia; E-Mails: amescic@fkit.hr (A.M.); prekupec@fkit.hr (S.K.); 2 Pharmaceutical Biochemistry, School of Pharmaceutical Sciences, University of Geneva, Quai Ernest-Ansermet 30, CH-1211 Geneva 4, Switzerland; E-Mails: Ivana.Novakovic@unige.ch (I.N.); Leonardo.Scapozza@unige.ch (L.S.); 3 Department of Pharmaceutical Chemistry, Faculty of Pharmacy, University of Sarajevo, Zmaja od Bosne 8, BIH-71 000 Sarajevo, Bosnia and Herzegovina; E-Mails: amkooo@hotmail.com (A.O.); dzavrsnik@yahoo.com (D.Z.); 4 Center for Radiopharmaceutical Sciences, ETH Zurich (Swiss Federal Institute of Technology), Wolfgang-Pauli Strasse 10, CH-8093 Zurich, Switzerland; E-Mails: ursinamuller@gmail.com (U.M.); simon.ametamey@pharma.ethz.ch (S.M.A.)

**Keywords:** acyclic nucleoside analogues, 5-substituted pyrimidines, herpes simplex virus type 1 thymidine kinase (HSV-1 TK), positron emission tomography (PET)

## Abstract

The efficient syntheses of 5-(2-hydroxyethyl)- and 5-(3-hydroxypropyl)-substituted pyrimidine derivatives bearing 2,3-dihydroxypropyl, acyclovir-, ganciclovir- and penciclovir-like side chains are reported. A synthetic approach that included the alkylation of an *N*-anionic-5-substituted pyrimidine intermediate (method A) provided the target acyclonucleosides in significantly higher overall yields in comparison to those obtained by method B using sylilation reaction. The phosphorylation assays of novel compounds as potential substrates for thymidine kinase of herpes simplex virus type 1 (HSV-1 TK) showed that solely pyrimidine 5-substituted acyclonucleosides with a penciclovir-like side chain acted as a fraudulent substrates of HSV-1 TK. Moreover, the uracil derivative with penciclovir-like side chain with less bulky 2-hydroxyethyl substituent at C-5 proved to be a better substrate than the corresponding one with a 3-hydroxypropyl substituent. Therefore, this acyclonucleoside was selected as a lead compound for the development of a positron emission tomography HSV-1 TK activity imaging agent.

## 1. Introduction

**Figure 1:**
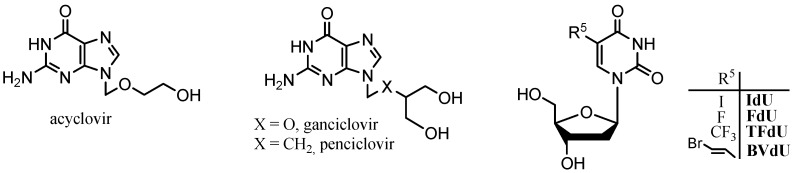
Acyclic nucleoside analogues and 2'-deoxyuridines as chemotherapeutic agents.

Nucleoside analogues have acquired an important role as therapeutic agents in the field of chemotherapy on account of their extensive biological activities [1,2]. Thereby, modified nucleosides and nucleobases are a pharmacologically diverse family, which includes anticancer compounds, antiviral agents, and immunosuppressive molecules. Interest in acyclic nucleosides began in the mid-1970s when acyclovir was first reported as a potent anti-herpes drug [3,4]. Many variations, both of the acyclic glycone and of the heterocyclic base, have been described [5,6]. Antiviral nucleoside analogues are known to localize selectively in herpes simplex virus (HSV)-infected cells because of monophosphorylation catalyzed by virus-encoded thymidine kinase (TK) [7]. Among these, acyclovir, ganciclovir and penciclovir are reported to be potent against HSV types 1 and 2 [8,9] (Figure 1).

In another area, a great number of 5-substituted uracil derivatives, especially 2'-deoxyuridines, have been investigated for the experimental and clinical treatment of neoplastic and viral diseases (Figure 1). The selective monophosphorylation of antiviral nucleoside analogues by virus-encoded thymidine kinase has been exploited in gene therapy of cancer [10]. Gene therapy offers promising new treatment modalities in oncology and other diseases [11]. The suicide gene, HSV-1 TK, has already been successfully employed in a variety of tumour models, both *in vitro* and *in vivo* [12,13]. A prodrug with low toxicity becomes toxic only in cells where the HSV-1 TK gene is expressed, thus resulting in the selective killing of transfected cells through termination of DNA synthesis. Phosphorylation of radioactive-labelled prodrugs, usually derivatives of acyclonucleosides which are selective anti-herpes agents, leads to metabolites trapped within the infected tumour cells, and the resulting accumulation of radioactivity allows monitoring of the enzyme activity with positron emission tomography (PET) [14,15,16]. A striking difference is that while all pyrimidine nucleoside analogues show low, if any, bystander killing effects, purine derivatives such as ganciclovir are endowed with bystander killer effect [17].

As found earlier, pyrimidine nucleoside analogues in which the acyclic sugar moiety is attached at the C-6 rather than at the N-1 position have the potential for the development as a new PET imaging probe. Therefore, thymines with 6-(2,3-dihydroxypropyl), 6-(1,3-dihydroxyisobutyl) and 6-(1,3-dihydroxyisobutenyl) side chains have been developed as tracer molecules for monitoring HSV-1 TK expression by means of PET [18,19,20]. Although the *N*-methylated thymine derivative, *N*-Me-[18F]FHBT, bearing a 1,3-dihydroxyisobutyl side-chain at the C-6 position was not superior to the purine analogue [18F]FHBG, an established PET reporter probe for imaging HSV-1 TK expression, *N*-Me-[18F]FHBT clearly showed the feasibility of its use as a PET probe to monitor HSV-1 TK gene expression *in vivo* [21].

**Figure 2:**
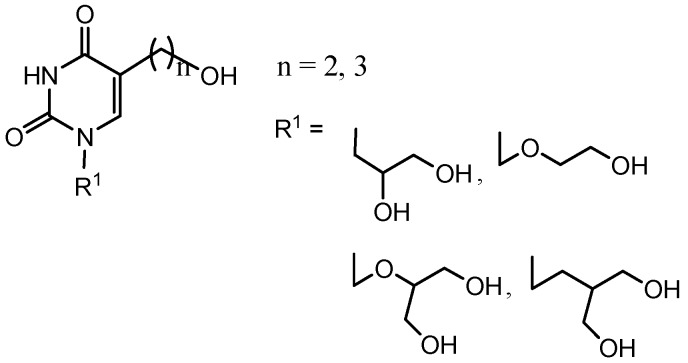
Novel 5-(2-hydroxyalkyl)-substituted uracil derivatives bearing 2,3-dihydroxy-propyl (**19**), acyclovir- (**21**), ganciclovir- (**23**) and penciclovir-like (**31** and **32**) side chains.

In an effort to further explore the pyrimidine scaffold as a putative starting point for the development of new fraudulent substrates of HSV-1 TK for non-invasive imaging of HSV-1 TK gene expression with improved pharmacodynamic and pharmacokinetic profile, we have synthesized a series of novel 5-substituted acyclic nucleoside analogues. Here we report on the synthesis of target 5-(2-hydroxyalkyl)-substituted uracil derivatives bearing 2,3-dihydroxypropyl (**19**), acyclovir- (**21**), ganciclovir- (**23**) and penciclovir-like (**31** and **32**) substituents at N-1 position of the pyrimidine ring (Figure 2). In order to evaluate target nucleoside mimetics **19**, **21**, **23**, **31** and **32** as substrates for HSV-1 TK, molecular modelling and phosphorylation assays of these compounds were performed.

## 2. Results and Discussion

### 2.1. Chemistry

**Scheme 1:**
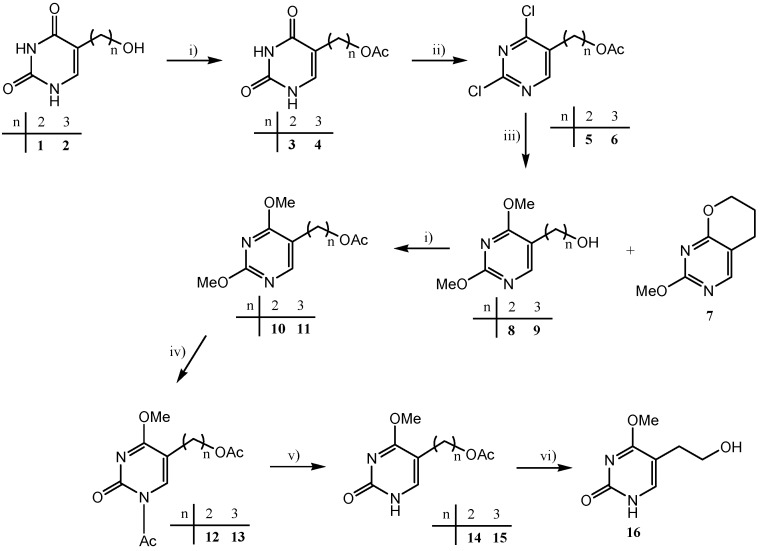
Synthesis of the precursors **10** and **14**–**16** for *N*-alkylation.

**Scheme 2:**
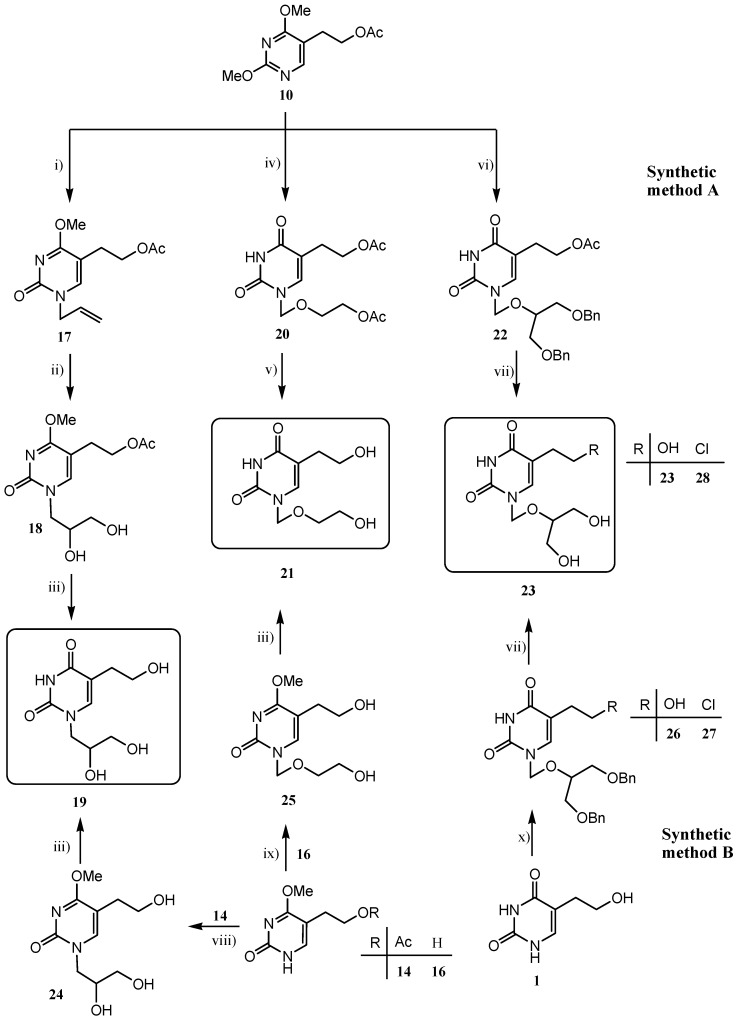
Synthesis of 5-(2-hydroxyethyl)uracil derivatives with 2,3-dihydroxypropyl (**19**), acyclovir- (**21**) and ganciclovir-like (**23**) side chain.

**Scheme 3:**
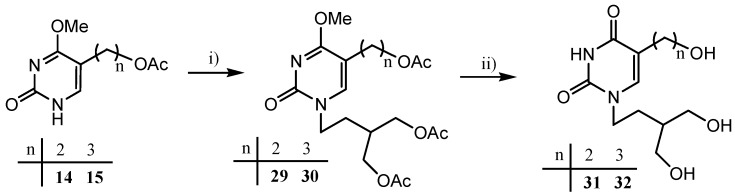
Synthesis of 5-(2-hydroxyalkyl)uracil derivatives **31** and **32** with penciclovir-like side chains.

5-(2-Hydroxyalkyl)uracil derivatives with 2,3-dihydroxypropyl (**19**), acyclovir- (**21**), ganciclovir- (**23**) and penciclovir-like (**31** and **32**) side chains were prepared from the corresponding uracil derivatives **10** and **14**–**16** by the methods outlined in Scheme 1, Scheme 2, Scheme 3. The synthesis of compounds **10** and **14**–**16**, as precursors for *N*-alkylation, was performed in excellent yields (80–99%) (Scheme 1). 5-(2-Hydroxyethyl)- and 5-(3-hydroxypropyl)-substituted uracils **1** and **2** served as starting materials and their syntheses were reported earlier [22,23,24]. The synthesis of 5-substituted 2,4-dimethoxypyrimidine derivatives **10** and **11** was carried out by acetylation, chlorination at the positions C-2 and C-4 with POCl_3_, methoxylation with sodium methoxide and repeated acetylation [25] (Scheme 1).

*Reagents and conditions*: (i) acetic anhydride, pyridine, rt, 1 h; (ii) POCl_3_, *N*,*N*-diethylaniline, pyridine, reflux, 1 h; (iii) NaOMe/MeOH, rt, 20 h; (iv) AcCl, rt, 20 h; (v) silica gel column chromatography; (vi) NaOMe/MeOH, rt, 1 h.

Selective 2-dealkylation of 5-(acetoxyalkyl)-2,4-dimethoxypyrimidines [26] **10** and **11** in non-aqueous acid gave the corresponding 4-methoxypyrimidin-2-one derivatives **14** and **15**. Treatment of the acetyl protected 2,4-dimethoxypyrimidines **10** and **11** with acetyl chloride afforded *N*-acetylated 4-methoxypyrimidin-2-one derivatives **12** and **13**, which after *N*-deprotection and purification using column chromatography on silica gel yielded **14** and **15** (Scheme 1). Final deacetylation of **14** gave 4-methoxypyrimidin-2-one **16** with a 2-hydroxyethyl substituent at C-5.

Further synthetic steps in the preparation of target compounds **19**, **21** and **23** involved *N*-alkylation applied by two synthetic methods (A and B, Scheme 2, Table 1). In order to ensure the introduction of the substituent into position N-1 of the pyrimidine ring, the Hilbert-Johnson reactions [27] were applied in synthetic method A. These reactions involved the treatment of 5-(2-acetoxyethyl)-2,4-dimethoxypyrimidine derivative **10** with alkyl halides. Allyl bromide and 1,3-dibenzyloxy-2-chloromethoxypropane [28] were used as agents for the introduction of 2,3-dihydroxypropyl (in **19**) and ganciclovir-like side chain (in **23**), respectively. Furthermore, the treatment of 1,3-dioxolane with acetyl bromide gave (2-acetoxyethoxy)methyl bromide [29] as a readily distilled colourless oil, which served as the alkylating reagent for the introduction of the acyclovir-like side chain (in **21**). Unlike alkylation with allyl bromide which gave *N*-1-substituted 4-methoxypyrimidine **17** in 73% yield, alkylation using (2-acetoxyethoxy)methyl bromide and 1,3-dibenzyloxy-2-chloromethoxypropane was accompanied by 4-demethylation and the corresponding *N*-1-substituted pyrimidin-2,4-dione derivatives **20** and **22** were isolated in moderate yield (43% and 36%, respectively).

*Reagents and conditions*: (i) allyl bromide; (ii) NaClO_3_, OsO_4_; (iii) 1 M NaOH, rt, 20 h; (iv) (2-acethoxyethoxy)methyl bromide, Na_2_CO_3_, rt, 24 h; (v) NaOMe/MeOH, rt, 20 h; (vi) 1,3-dibenzyloxy-2-chloromethoxypropane, K_2_CO_3_, CH_2_Cl_2_, rt, 24 h; (vii) BCl_3_, CH_2_Cl_2_, 3 h; (viii) NaH, DMF, 1-chloro-2,3-dihydroxypropane; (ix) BSA, CH_3_CN, dioxolane, KI, TMSCl, rt, 48 h; (x) 1. HMDS, TMSCl; 2. 1,3-dibenzyloxy-2-chloromethoxypropane, TBAI, CH_3_CN, rt, 24 h.

**Table 1:** An overview of the yields in synthetic method A and B for target compounds **19**, **21** and **23**.

**Synthetic method A**		**19**	**21**	**23**
		**Yields**		
1.	Acetylation	98%	98%	98%
2.	Chlorination	99%	99%	99%
3.	Methoxylation	95%	95%	95%
4.	Acetylation	90%	90%	90%
5.	Alkylation/Hydroxylation	73%/50%	43%	36%
6.	Deprotection	39%	74%	67%
	Overall yield:	12%	26%	20%
**Synthetic method B**		**19**	**21**	**23**
		**Yields**		
1.	Acetylation	98%	98%	-
2.	Chlorination	99%	99%	-
3.	Methoxylation	95%	95%	-
4.	Acetylation	90%	90%	-
5.	Demethoxylation	80%	80%	-
6.	Deacetylation	-	93%	-
7.	Silylation/Akylation	75%	25%	8%
8.	Deprotection	37%	26%	65%
	Overall yield:	18%	4%	5%

*cis*-Dihydroxylation of *N*-1-allyl pyrimidine derivative **17** with sodium chlorate in the presence of osmium tetroxide afforded *N*-1-(2,3-dihydroxypropyl)pyrimidine derivative **18** which after the 4-demethylation and deacetylation with sodium hydroxide yielded the desired 5-(2-hydroxyethyl)pyrimidine **19** bearing a 2,3-dihydroxypropyl group at N-1. The overall yield of this method was 12%. The deacetylation of the side chains at C-5 and N-1 of **20** under basic conditionsafforded the 5-(2-hydroxyethyl)pyrimidine **21** with the best overall yield of 26%. Similarly, both deacetylation in C-5 and debenzylation in N-1 side chains of **22** using boron trichloride gave the 5-(2-hydroxyethyl)-substituted pyrimidine **23** with a ganciclovir-like side chain at N-1.

In search for a more efficient synthesis of **19**, an alternate method B was applied using sodium hydride as deprotonating agent and thus the uracil salt obtained *in situ* reacted with 1-chloro-2,3-dihydroxypropane to give trihydroxy 4-methoxypyrimidin-2-one **24**. Deprotection of **24** yielded target the trihydroxypyrimidin-2,4-dione **19**. In comparison to synthetic method A this method for preparation of **19** is more efficient and the use of the highly poisonous reagent osmium tetroxide for *cis*-hydroxylation was avoided. Similarly, compounds **21** and **23** were also prepared by silylation reactions (synthetic method B). Since silylation of 5-(2-hydroxyethyl)uracil (**1**) using *N,O*-bis-(trimethysilyl)acetamide (BSA) was unsuccessful, pyrimidin-2-one **16** was used for *N*-alkylation. Trimethylsilylation of **16** [26] using BSA and *in situ* coupling employing trimethylchlorosilane, potassium iodide and 1,3-dioxolane in dry acetonitrile was performed, and the expected product **21** was obtained after hydrolysis of **25** with sodium hydroxide (Scheme 2). Furthermore, compound **23** was prepared directly from 5-(2-hydroxyethyl)uracil (**1**) by the silylation of **1** and *in situ* reaction of O-persilylated **1** with 1,3-dibenzyloxy-2-chloromethoxypropane to give *N*-1-alkylated pyrimidine **26** in 8% yield (method B). The removal of the benzyl protecting groups in **26** proceeded smoothly and led to target compound **23**.

For the introduction of a penciclovir-like side chain at N-1 position of the pyrimidine ring, 4-acetoxy-(3-acetoxymethyl)butyl iodide [30,31], prepared from a mesylate and the related bromide, was used. Since neither direct Hilbert-Johnson-type reaction of 2,4-dimethoxypyrimidine derivative **10** with both alkyl halides and mesylate did not yield the corresponding pyrimidine acyclonucleoside, an alternative method using 4-methoxypyrimidin-2-one derivatives **14** and **15** was applied.

Thus, the reaction of potassium salts of **14** and **15** with 4-acetoxy-(3-acetoxymethyl)butyl iodide led to the target 5-(2-hydroxyethyl)- **31** and 5-(3-hydroxypropyl)pyrimidine **32** bearing a penciclovir-like side chain (Scheme 3).

*Reagents and conditions*: (i) 4-Acetoxy-(3-acetoxymethyl)butyl iodide, K_2_CO_3_, DMF, 60 °C, 20 min; (ii) 1 M NaOH, rt, 20 h.

### 2.2. *In Vitro* Validation of Compounds **19**, **21**, **23**, **31** and **32** as Substrates of HSV-1 TK and hTK

**Figure 3:**
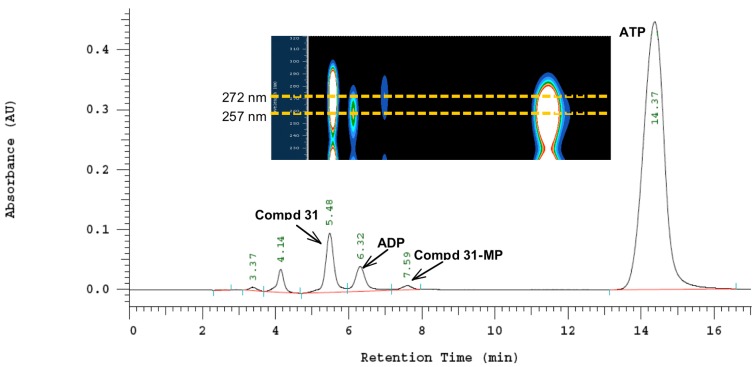
HPLC and DAD/HPLC chromatograms of the reaction of **31** and HSV-1 TK.

**Figure 4:**
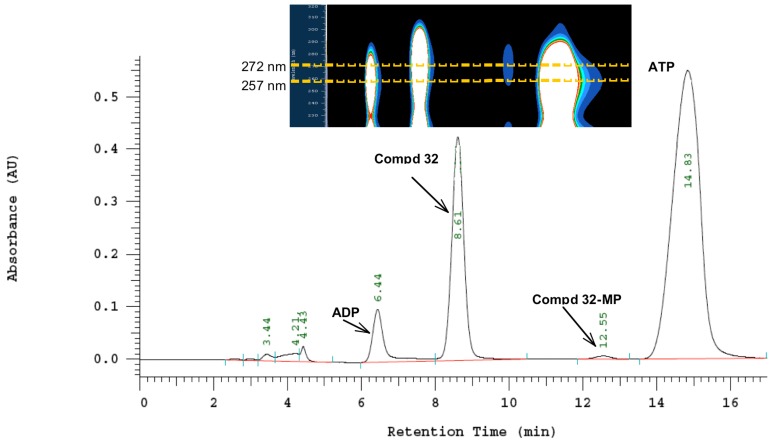
HPLC and DAD/HPLC chromatograms of the reaction of **32** and HSV-1 TK.

To determine whether compounds **19**, **21**, **23**, **31** and **32** are substrates of HSV-1 TK or human TK, phosphorylation assays were performed by incubation of deoxythymidine (dT) and target compounds with recombinant HSV-1 TK and human TK (hTK) according to a previously published protocol [32]. The monophosphorylation was monitored qualitatively by the formation of new peaks corresponding to adenosine diphosphate (ADP), monophosphate derivatives of tested compounds and thymidine monophosphate (dTMP). Results of phosphorylation assays for **19**, **21** and **23** indicated that these compounds are not phosphorylated by either HSV-1 TK or human TK (see Supplementary Materials). While acyclonucleosides containing dihydroxypropyl (**19**), acyclovir- (**21**) and ganciclovir-like side chain (**23**) were not phosphorylated, both 5-substituted acyclic pyrimidines **31** and **32** with a penciclovir-like side chain showed to be substrates of HSV-1 TK. Furthermore, small alteration in structures of **23**, such as replacement of oxygen in the (1,3-dihydroxy-2-propoxy)methyl side chain at N-1 with a methylene group (compound **31**), has an impact on phosphorylation reaction of these compounds. We can assume that the electron donating oxygen has an influence on the interaction of **23** and HSV-1 TK disabling the enzyme to phosphorylate fraudulent substrate **23** with a ganciclovir-like side chain. Therefore, for compounds **31** and **32** while using viral HSV-1 TK, the formation of monophosphate derivatives **31-MP** (Figure 3) and **32-MP** (Figure 4) as well as ADP were detected, confirming that **31** and **32** are substrates of viral HSV-1 TK. However, the size of the alkyl substituent at C-5 exhibited some effect on phosphorylation. Thus, compound **31** with a less bulky 2-hydroxyethyl substituent at C-5 had a higher ADP/ATP ratio compared to compound **32**, thus proving to be a better substrate than **32**. Furthermore, formation of **31-MP** and **32-MP** was proven by extracting the max. absorbance wavelength of the new appearing peaks which corresponds to the max. absorbance wavelength of compounds **31** and **32** (Figure 3, Figure 4). No phosphorylation was observed for compounds **31** and **32** when human TK was used, indicating that these two compounds are not substrates for hTK. 

**Figure 5:**
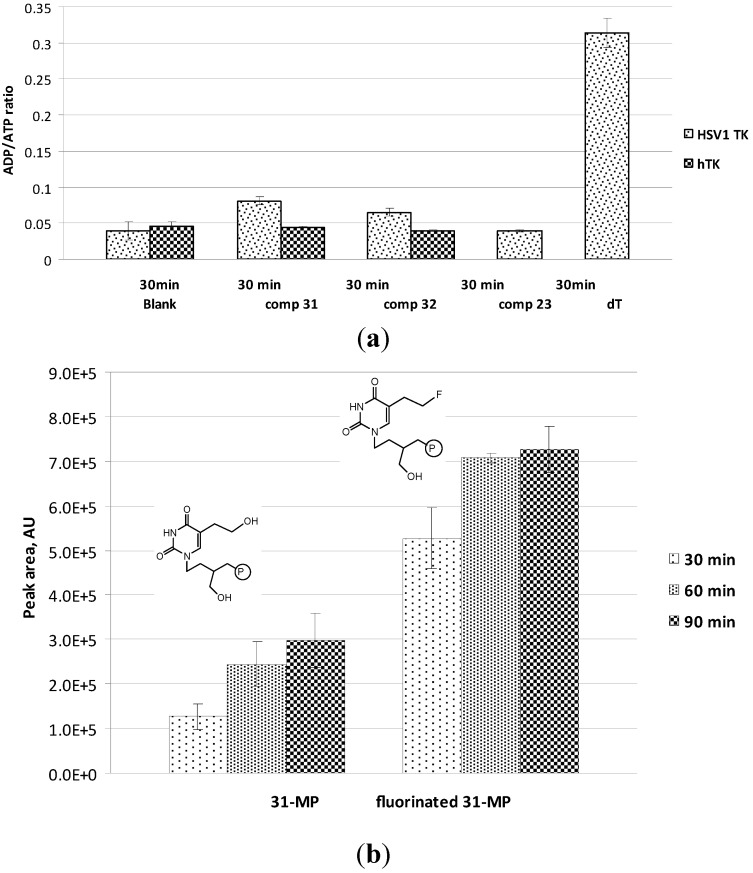
(**a**) Phosphorylation pattern of 1 mM of compounds **31** and **32**, by HSV-1 TK and hTK, and of compound **23** and dT by HSV-1 TK. Blank refers to the control experiment performed in presence of the enzyme and in absence of the compound; (**b**) Time course for conversion of **31** and its fluorinated analogue to corresponding monophosphated derivatives.

The phosphorylation patterns of compounds **31** and **32** by HSV-1 TK and hTK are presented in Figure 5a. No significant difference in ADP/ATP ratio was noticed in phosphorylation assays with hTK.

Phosphorylation reaction of compound **31** and its fluorinated structural analogue, in which hydroxyl group in C-5 substituent is replaced with fluorine, was performed at 30, 60 and 90 min of incubation (Figure 5b). It is evident that formation of monophosphated derivatives for both compounds was increased over time. By comparison of calculated values for monophosphated derivatives which were formed at different times (30, 60 and 90 min), we can estimate that approximately 40% of **31-MP** was formed in 30 min and majority of **31-MP** (~80%) in 60 min of incubation. On the contrary, fluorinated derivative gave majority of corresponding monophosphate (~70%) already in 30 min and was almost completely monophosphorylated in 60 min. The synthesis of fluorinated derivative of **31** and *in vivo* results on small animal PET studies of ^18^F labeled compound **31**, HHB-5-[18F]FEP, will be presented elsewhere.

### 2.3. Cellular Activity Evaluation

Compounds **17**–**32** were evaluated for their cytotoxic activity against normal human fibroblasts (WI38). The results indicated that the target compounds exhibited no cytotoxic effects against normal human fibroblasts up to 100 µM.

## 3. Experimental

### 3.1. General

Melting points (uncorrected) were determined with a Kofler micro hot-stage (Reichert, Wien, Austria). Precoated Merck (Darmstadt, Germany) silica gel 60F-254 plates were used for thin layer chromatography and the spots were detected under UV light (254 nm). Column chromatography was performed using Fluka (Buchs, Switzerland) silica gel (0.063–0.2 mm); glass columns were slurry-packed under gravity. ^1^H- and ^13^C-NMR spectra were acquired on a Bruker 300 MHz NMR spectrometer (Bruker Biospin, Rheinstetten, Germany) All data were recorded in DMSO-*d*_6_ at 298 K. Chemical shifts were referenced to the residual solvent signal of DMSO at δ 2.50 ppm for ^1^H and δ 39.50 ppm for ^13^C. Individual resonances were assigned on the basis of their chemical shifts, signal intensities, multiplicity of resonances and H−H coupling constants. Mass spectra were recorded on an Agilent 6410 instrument (Agilent Technologies, Wilmington, DE, USA) equipped with an electrospray interface and triple quadrupole analyzer (LC/MS/MS). High performance liquid chromatography was performed on an Agilent 1100 series system with UV detection (photodiode array detector) using a Zorbax C18 reverse-phase analytical column (2.1 × 30 mm, 3.5 µm). All compounds used for biological evaluation showed >95% purity in this HPLC system.

### 3.2. Procedures for the Preparation of Compounds

5-(2-Hydroxyethyl)pyrimidin-2,4-dione (**1**), 5-(3-hydroxypropyl)pyrimidin-2,4-dione (**2**), 5-(2- acetoxyethyl)pyrimidin-2,4-dione (**3**), 5-(2-acetoxyethyl)-2,4-dichloropyrimidine (**5**), 5-(2-hydroxy-ethyl)-2,4-dimethoxypyrimidine (**8**), 5-(2-acetoxyethyl)-2,4-dimethoxypyrimidine (**10**), 5-(2-acetoxy-ethyl)-4-methoxypyrimidin-2-one (**14**) and 5-(2-hydroxyethyl)-4-methoxypyrimidin-2-one (**16**) were synthesized in accord with original procedures given in the literature [22,23,24,25,26].

*5-(3-Acetoxypropyl)pyrimidin-2,4-dione* (**4**). The mixture of compound **2** (30 g, 0.18 mol) and acetic anhydride (138 mL, 1.46 mol) in anhydrous pyridine (327 mL) was stirred at room temperature for 1 h. The reaction was quenched with water and the mixture was evaporated to dryness. After column chromatography (CH_2_Cl_2_–CH_3_OH = 8:1) white crystals of compound **4** were isolated (35.14 g, 94%, m.p.: 224–227 °C). ^1^H-NMR: (δ) 11.00 (1H, s, NH), 10.64 (1H, s, NH), 7.23 (1H, s, H-6), 3.97 (2H, t, H-3', *J* = 6.5 Hz), 2.21 (2H, t, H-1', *J* = 7.3 Hz), 2.00 (3H, s, COCH_3_), 1.77–1.64 (2H, m, H-2') ppm. ^13^C-NMR: (δ) 170.9 (COCH_3_), 164.9 (C-4), 151.8 (C-2), 138.6 (C-6), 111.5 (C-5), 63.7 (C-3'), 27.5 (C-2'), 23.2 (C-1'), 21.2 (COCH_3_) ppm. MS (ESI): *m/z* = 213.1 ([*M*+H]^+^).

*5-(3-Acetoxypropyl)-2,4-dichloropyrimidine* (**6**). The mixture of compound **4** (34 g, 0.16 mol), POCl_3_ (320 mL) and *N*,*N*-diethylaniline (32 mL) was refluxed for 1 h. The solvent was evaporated to dryness, the residue was diluted with CCl_4_ (300 mL), washed twice with iced water and dried over MgSO_4_. After the removal of the solvent, compound **6** (34.87 g, 87%) was isolated as a yellow oil. ^1^H-NMR: (δ) 8.8 (1H, s, H-6), 4.11 (2H, t, H-3', *J* = 6.3 Hz), 2.81 (2H, t, H-1', *J* = 7.7 Hz), 1.97 (2H, m, H-2'), 2.01 (3H, s, COCH_3_) ppm. ^13^C-NMR: (δ) 170.1 (COCH_3_), 161.2 (C-4), 161.1 (C-2), 156.5 (C-6), 132.4 (C-5), 62.8 (C-3'), 26.9 (C-2'), 25.6 (C-1'), 20.4 (COCH_3_) ppm. MS (ESI): *m/z* = 250.1 ([*M*+H]^+^).

*2-Methoxy-6,7-dihydro-5H-pyrano[2,3-d]pyrimidine* (**7**) *and 2,4-dimethoxy-5-(3-hydroxypropyl)-pyrimidine* (**9**). To a solution of sodium (16.7 g, 0.72 mol) in anhydrous methanol (703 mL) compound **6** (34.87 g, 0.14 mmol) was added. The reaction mixture was stirred at room temperature for 20 h, neutralized with ether saturated with gaseous HCl at 0 °C. The solvent was evaporated and CHCl_3_ (560 mL) was added. The mixture was stirred at room temperature for 20 h and filtered. The filtrate was dried over MgSO_4_ and evaporated to dryness. After column chromatography (CH_2_Cl_2_–CH_3_OH = 35:1) compounds **7** (9.66 g, 42%, m.p.: 68–70 °C) and **9** (5.29 g, 19%) were isolated.

**7**: ^1^H-NMR: (δ) 8.15 (1H, s, H-6), 4.32 (2H, t, H-3', *J* = 5.2 Hz), 3.79 (3H, s, OCH_3_), 2.65 (2H, t, H-1', *J* = 6.4 Hz), 1.92–1.88 (2H, m, H-2') ppm. ^13^C-NMR: (δ) 168.5 (C-4), 164.4 (C-2), 159.2 (C-6), 109.2 (C-5), 68.3 (C-3'), 54.7 (OCH_3_), 21.0 (C-2'), 21.4 (C-1') ppm. MS (ESI): *m/z* = 167.1 ([*M*+H]^+^).

**9**: ^1^H-NMR: (δ) 8.1 (1H, s, H-6), 4.47 (1H, t, OH, *J* = 5.2 Hz), 3.92 (3H, s, OCH_3_), 3.87 (3H, s, OCH_3_), 3.41 (2H, q, H-3', *J* = 6.0 Hz), 2.47 (2H, t, H-1', *J* = 7.7 Hz), 1.65 (2H, k, H-2', *J* = 7.0 Hz) ppm. ^13^C-NMR: (δ) 169.3 (C-4), 164.0 (C-2), 157.5 (C-6), 115.1 (C-5), 60.5 (C-3’), 54.7 (OCH_3_), 54.1 (OCH_3_), 32.1 (C-2'), 22.9 (C-1') ppm. MS (ESI): *m/z* = 199.1 ([*M*+H]^+^).

*5-(3-Acetoxypropyl)-2,4-dimethoxypyrimidine* (**11**). To a stirred solution of compound **9** (5.2 g, 26.23 mmol) in dry pyridine (48 mL) acetic anhydride (20 mL, 26.4 mmol) was added and the stirring was continued for 1 h at room temperature. After the completion of reaction 15 mL of water was added. The solvent was evaporated and the residue was purified by column chromatography (CH_2_Cl_2_–CH_3_OH = 30:1) to yield compound **11** as a yellow oil (6.1 g, 97%). ^1^H-NMR: (δ) 8.12 (1H, s, H-6), 3.98 (2H, t, H-3', *J* = 6.5 Hz), 3.91 (3H, s, OCH_3_), 3.86 (3H, s, OCH_3_), 2.49 (2H, t, H-1', *J* = 7.8 Hz), 2.0 (3H, s, COCH_3_), 1.81 (2H, k, H-2', *J* = 7.0 Hz) ppm. ^13^C-NMR: (δ) 170.85 (COCH_3_), 169.29 (C-4), 162.9 (C-2), 157.7 (C-6), 114.3 (C-5), 63.6 (C-3'), 54.7 (OCH_3_), 54.2 (OCH_3_), 27.8 (C-2'), 22.9 (C-1'), 21.1 (COCH_3_) ppm. MS (ESI): *m/z* = 241.1 ([*M*+H]^+^).

*5-(3-Acetoxypropyl)-4-methoxypyrimidin-2-one* (**15**). To a dried two-necked flask compound **11** (3.23 g, 13.44 mmol) and acetyl chloride (26 mL, 0.36 mol) were added under argon atmosphere. The reaction mixture was stirred at room temperature for 20 h. After the reaction was complete, the solvent was evaporated at reduced pressure at 35 °C and coevaporated twice by the addition of toluene (6 mL). The residue was chromatographed (EtOAc–CH_3_OH = 20:1). During this step, the intermediate **13** was deacetylated to form a white crystalline product **15** (1.79 g, 59%, m.p.: 146–149 °C). Compound **4** was also isolated (966 mg, 34%). ^1^H-NMR: (δ) 11.14 (1H, s, NH), 7.50 (1H, s, H-6), 3.97 (2H, t, H-3', *J* = 6.5 Hz), 3.83 (3H, s, OCH_3_), 2.32 (2H, t, H-1', *J* = 7.4 Hz), 1.99 (3H, s, COCH_3_), 1.75 (2H, k, H-2', *J* = 6.7 Hz) ppm. ^13^C-NMR: (δ) 170.9 (COCH_3_), 170.8 (C-4), 156.6 (C-2), 143.1 (C-6), 105.8 (C-5), 63.6 (C-3'), 54.1 (OCH_3_), 27.8 (C-2'), 22.8 (C-1'), 21.1 (COCH_3_) ppm. MS (ESI): *m/z* = 227.2 ([*M*+H]^+^).

*5-(2-Acetoxyethyl)-N-1-allyl-4-methoxypyrimidin-2-one* (**17**). *Method A*: A solution of compound **10** (375 mg, 1.66 mmol) and allyl bromide (0.15 mL, 1.73 mmol) was heated under reflux for 8 h. After evaporation of the solvent and column chromatography (ethyl acetate:CH_3_OH = 15:1) compound **17** was obtained as a colourless oil (305.3 mg, 73%). ^1^H-NMR: (δ) 7.78 (1H, s, H-6), 5.90 (1H, m, H-2"), 5.16 (1H, dd, H-3", *J*_1_ = 1.3 Hz, *J*_2_ =10.3 Hz), 5.06 (1H, dd, H-3", *J*_1_ = 1.4 Hz, *J*_2_ =17.2 Hz), 4.08 (2H, t, H-2', *J* = 6.6 Hz), 4.36 (2H, d, H-1", *J* = 5.4 Hz), 3.83 (3H, s, OCH_3_), 2.57 (2H, t, H-1', *J* = 6.5 Hz), 1.95 (3H, s, COCH_3_) ppm. ^13^C-NMR: (δ) 170.7 (COCH_3_), 170.1 (C-4), 155.6 (C-2), 147.5 (C-6), 103.6 (C-5), 133.7 (C-2"), 117.8 (C-3"), 62.6 (C-2'), 54.5 (OCH_3_), 51.1 (C-1"), 26.0 (C-1'), 21.1 (COCH_3_) ppm. MS (ESI): *m/z* = 253.1 ([*M*+H]^+^).

*5-(2-Acetoxyethyl)-N-1-(2,3-dihydroxypropyl)-4-methoxypyrimidin-2-one* (**18**). *Method A*: OsO_4_ (1 mg, 0.004 mmol) was added to a solution of compound **17** (203.5 mg, 0.81 mmol), followed by addition of sodium chlorate (103 mg, 0.97 mmol) dissolved in water (2.5 mL). The reaction mixture was stirred at room temperature for 3 days. The mixture was then filtered through Celite and applied to an Amberlite IR 4 B column using water as eluens. The product was evaporated to dryness and applied on a Dowex (100–200 mesh, H^+^-form) column and eluted with water. The eluate was evaporated under reduced pressure and the residue was purified by column chromatography (CH_2_Cl_2_–CH_3_OH = 10:1) to yield compound **18** as a yellow oil (115.5 mg, 50%). ^1^H-NMR: (δ) 7.71 (1H, s, H-6), 4.95 (1H, d, OH, *J* = 5.5 Hz), 4.95 (1H, t, OH, *J* = 5.4 Hz), 4.02–4.11 (3H, m, H-2', H-3"), 3.84 (3H, s, OCH_3_), 3.70–3.78 (1H, m, H-2"), 3.42–3.48 (1H, m, H-3"), covered with water (H-1"), 2.58 (2H, t, H-1', *J* = 6.5 Hz), 1.98 (3H, s, COCH_3_) ppm. ^13^C-NMR: (δ) 170.8 (COCH_3_), 170.0 (C-4), 156.0 (C-2), 149.2 (C-6), 102.4 (C-5), 69.2 (C-2"), 64.1 (C-3"), 62.6 (C-2'), 54.4 (OCH_3_), 53.0 (C-1"), 25.9 (C-1'), 21.1 (COCH_3_) ppm. MS (ESI): *m/z* = 287.1 ([*M*+H]^+^). 

*5-(2-Hydroxyethyl)-N-1-(2,3-dihydroxypropyl)pyrimidin-2,4-dione* (**19**). *Method A*: Compound **18** (92.4 mg, 0.32 mmol) was dissolved in 1 M NaOH (8 mL) and the mixture was stirred at room temperature for 20 h. Then, it was neutralized by the addition of acetic acid. After evaporation of the solvents and column chromatography (CH_2_Cl_2_–CH_3_OH = 5:1) white crystals of compound **19** were obtained (29 mg, 39%, m.p.: 95–98 °C). 

*Method B*: Compound **24** (164 mg, 0.67 mmol) was dissolved in 1 M NaOH (20 mL) and the mixture was stirred at room temperature for 20 h and neutralized by the addition of HCl. After evaporation of the solvent and column chromatography (CH_2_Cl_2_–CH_3_OH = 5:1), compound **19** was obtained (57.7 mg, 37%). ^1^H-NMR: (δ) 11.18 (1H, bs, NH), 7.36 (1H, s, H-6), 4.95 (1H, d, OH, *J* = 5.5 Hz), 4.69 (1H, t, OH, *J* = 5.6 Hz), 4.53 (1H, t, OH, *J* = 5.6 Hz), 3.89 (1H, dd, H-3", *J_1_* = 3.8 Hz, *J_2_* =13.3 Hz), 3.64–3.72 (1H, m, H-2"), 3.45 (2H, q, H-2', *J* = 6.7 Hz), 3.36–3.41 (3H, m, H-3", H-1"), 2.29–2.34 (2H, m, H-1') ppm. ^13^C-NMR: (δ) 164.6 (C-4), 151.5 (C-2), 144.3 (C-6), 109.3 (C-5), 69.6 (C-2"), 64.1 (C-3"), 60.0 (C-2'), 51.4 (C-1"), 30.4 (C-1') ppm. MS (ESI): *m/z* = 231.1 ([*M*+H]^+^). Anal. Calcd for C_9_H_14_N_2_O_5_: C, 46.95; H, 6.13; N, 12.17. Found: C, 46.90; H, 6.15; N, 12.22.

*N-1-[(2-Acetoxyethoxy)methyl]-5-(2-acetoxyethyl)pyrimidin-2,4-dione* (**20**). *Method A*: To a stirred solution of compound **10** (47.2 mg, 0.21 mmol) and Na_2_CO_3_ (21.9 mg, 0.2 mmol) in anhydrous CH_2_Cl_2_ (1 mL), (2-acethoxyethoxy)methyl bromide [prepared *in situ* by adding acetyl bromide (0.02 mL, 0.2 mmol) to 1,3-dioxolane (0.01 mL, 0.2 mmol)] was added. The reaction mixture was stirred overnight at room temperature and evaporated to dryness. After column chromatography (CH_2_Cl_2_–CH_3_OH = 20:1) compound **20** was isolated as a crude product (28.2 mg, 43%, m.p.: 53–55 °C). ^1^H-NMR: (δ) 11.41 (1H, bs, NH), 7.64 (1H, s, H-6), 5.09 (2H, s, H-1"), 4.06–4.17 (4H, m, H-3", H-4"), 3.69 (2H, t, H-2', *J* = 4.6 Hz), 2.70 (2H, t, H-1', *J* = 6.4 Hz), 1.99 (3H, s, COCH_3_), 1.98 (3H, s, COCH_3_) ppm. ^13^C-NMR: (δ) 170.2 (COCH_3_), 163.7 (C-4), 150.9 (C-2), 142.0 (C-6), 109.4 (C-5), 76.3 (C-1"), 66.7 (C-3"), 62.9 (C-4"), 61.9 (C-2’), 25.8 (C-1'), 20.6 (COCH_3_), 20.6 (COCH_3_) ppm. MS (ESI): *m/z* = 315.1 ([*M*+H]^+^).

*N-1-[(2-Hydroxyethoxy)methyl]-5-(2-hydroxyethyl)pyrimidin-2,4-dione* (**21**). *Method A*: To a solution of sodium (2.1 mg, 0.09 mmol) in anhydrous methanol (0.5 mL) compound **20** (27 mg, 0.09 mmol) dissolved in methanol (1 mL) was added. The reaction mixture was stirred at room temperature for 1 h and neutralized with 1 M HCl/ether. After evaporation to dryness and column chromatography (CH_2_Cl_2_–CH_3_OH = 5:1) compound **21** was isolated (14.6 mg, 74%) as a colourless oil.

*Method B*: Compound **25** (37 mg, 0.15 mmol) was dissolved in 1 M NaOH (10 mL) and the mixture was stirred at room temperature for 20 h. Then, it was neutralized by the addition of acetic acid. After evaporation of the solvents and column chromatography (ethyl acetate–CH_3_OH = 5:1) compound **21** was obtained (9.1 mg, 26%). ^1^H-NMR: (δ) 11.25 (1H, bs, NH), 7.52 (1H, s, H-6), 5.08 (2H, s, H-1"), 4.60 (2H, bs, OH), 3.45–3.50 (6H, m, H-2', H-3", H-4"), 2.35 (2H, t, H-1', *J* = 6.6 Hz) ppm. ^13^C-NMR: (δ) 164.5 (C-4), 151.5 (C-2), 142.0 (C-6), 111.0 (C-5), 77.0 (C-1"), 71.0 (C-3"), 60.5 (C-4"), 60.0 (C-2'), 30.3 (C-1') ppm. MS (ESI): *m/z* = 231.1 ([*M*+H]^+^). Anal. Calcd for C_9_H_14_N_2_O_5_: C, 46.95; H, 6.13; N, 12.17. Found: C, 46.99; H, 6.15; N, 12.12.

*5-(2-Acetoxyethyl)-N-1-[(1,3-dibenzyloxy-2-propoxy)methyl]pyrimidin-2,4-dione* (**22**). *Method A*: To a stirred solution of compound **10** (114 mg, 0.50 mmol) and K_2_CO_3_ (186.4 mg, 1.35 mmol) in anhydrous CH_2_Cl_2_ (3.7 mL), 1,3-dibenzyloxy-2-chloromethoxypropane (257.6 mg, 0.8 mmol) was added under argon atmosphere. The reaction mixture was stirred overnight at room temperature and after filtration evaporated to dryness. After column chromatography (CH_2_Cl_2_–CH_3_OH = 35:1) compound **22** was isolated as a colourless oil (87 mg, 36%). ^1^H-NMR: (δ) 11.38 (1H, s, NH), 7.63 (1H, s, H-6), 7.40–7.22 (10H, m, Ph), 5.2 (2H, s, H-1"), 4.46 (4H, s, H-5"), 4.03 (2H, t, H-2', *J* = 6.8 Hz), 3.99–3.94 (1H, m, H-3"), 3.57–3.40 (4H, m, H-4"), 2.44 (2H, t, H-1', *J* = 6.8 Hz), 1.98 (3H, s, COCH_3_) ppm. ^13^C-NMR: (δ) 170. 7 (COCH_3_), 164.2 (C-4), 151.5 (C-2), 143.9 (C-6), 138.7 (Ph), 128.7 (Ph), 127.9 (Ph), 127.8 (Ph), 109.7 (C-5), 77.2 (C-3"), 76.5 (C-1"), 72.7 (C-5"), 70.3 (C-4"), 62.4 (C-2'), 26.2 (C-1'), 21.1 (COCH_3_) ppm. MS (ESI): *m/z* = 483.2 ([*M*+H]^+^).

*5-(2-Hydroxyethyl)-N-1-[(1,3-dihydroxy-2-propoxy)methyl]pyrimidin-2,4-dione* (**23**). *Method A*: A solution of compound **22** (63 mg, 0.13 mmol) in anhydrous CH_2_Cl_2_ (5 mL) was cooled to −68 °C and 1 M solution of BCl_3_ in CH_2_Cl_2_ (0.45 mL, 0.45 mmol) was added under argon atmosphere. The reaction mixture was stirred at −68 °C for 3 h, quenched by the addition of CH_2_Cl_2_–CH_3_OH (1:1) solution (15 mL) and allowed to warm to room temperature. The solvent was then evaporated and the residue chromatographed (CH_2_Cl_2_–CH_3_OH = 25:1). Compound **23** was isolated as a white solid (22.7 mg, 67%, m.p.: 118–120 °C).

*Method B*: In the same way as described for **22**, the deprotection of **26** was performed. The reagents used were: compound **26** (106 mg, 0.24 mmol), anhydrous CH_2_Cl_2_ (5 mL) and BCl_3_ (0.7 mL, 0.7 mmol). After column chromatography (CH_2_Cl_2_–CH_3_OH = 10:1) compound **23** (40.9 mg, 65%) was isolated. ^1^H-NMR: (δ) 11.27 (1H, s, NH), 7.53 (1H, s, H-6), 5.16 (2H, s, H-1"), 4.64–4.59 (3H, m, 3×OH), 3.52–3.40 (7H, m, H-2', H-4", H-3") 2.35 (2H, t, H-1', *J* = 6.6 Hz) ppm. ^13^C-NMR: (δ) 164.5 (C-4), 151.4 (C-2), 142.1 (C-6), 110.7 (C-5), 80.9 (C-3"), 76.3 (C-1"), 61.4 (C-4"), 59.9 (C-2'), 30.3 (C-1') ppm. MS (ESI): *m/z* = 261.1 ([*M*+H]^+^). Anal. Calcd for C_10_H_16_N_2_O_6_: C, 46.15; H, 6.20; N, 10.76. Found: C, 46.10; H, 6.22; N, 10.72.

*5-(2-Hydroxyethyl)-N-1-(2,3-dihydroxypropyl)-4-methoxypyrimidin-2-one* (**24**). *Method B*: A mixture of compound **14** (194.4 mg, 0.91 mmol) and sodium hydride (90.9 mg, 2.27 mmol, 60% dispersion in oil) in DMF (6 mL) was stirred at 60 °C for 1 h. To the resulting suspension glycerol *α*-chlorohydrine (0.6 mL, 6.82 mmol) was added dropwise and the mixture was stirred at 70 °C overnight. The solvent was evaporated under reduced pressure and after column chromatography (CH_2_Cl_2_–CH_3_OH = 5:1) compound **24** (166.9 mg, 75%) was obtained. ^1^H-NMR: (δ) 7.63 (1H, s, H-6), 4.92 (1H, d, OH, *J* = 5.8 Hz), 4.71 (1H, t, OH, *J* = 5.8 Hz), 4.53 (1H, t, OH, *J* = 5.5 Hz), 4.05 (1H, dd, H-3", *J_1_* = 3.3 Hz, *J_2_* = 13.0 Hz), 3.83 (3H, s, OCH_3_), 3.69–3.71 (1H, m, H-2"), 3.5–3.4 (2H, m, H-2'), covered with water (H-3", H-1"), 2.40 (2H, t, H-1', *J* = 6.4 Hz) ppm. ^13^C-NMR: (δ) 170.2 (C-4), 156.9 (C-2), 148.8 (C-6), 103.6 (C-5), 69.3 (C-2"), 64.1 (C-3"), 60.1 (C-2'), 54.3 (OCH_3_), 53.0 (C-1"), 30.0 (C-1') ppm. MS (ESI): *m/z* = 245.1 ([*M*+H]^+^). 

*N-1-[(2-Hydroxyethoxy)methyl]-5-(2-hydroxyethyl)-4-methoxypyrimidin-2-one* (**25**). *Method B*: To a stirred solution of compound **16** (110 mg, 0.65 mmol) in acetonitrile (6 mL) under exclusion of moisture *N*,*O*-bis-trimethysilylacetamide (BSA: 0.8 mL, 3.2 mmol) was added. The mixture was stirred until a clear solution appeared (about 10 min). Dioxolane (0.12 mL, 1.66 mmol), KI (139 mg, 0.83 mmol) and TMSCl (0.1 mL) were added sequentially. The stirring was continued for 48 h at room temperature. The volatile materials were evaporated under reduced pressure and the residue was purified using column chromatography (CH_2_Cl_2_–CH_3_OH = 5:1) to give compound **25** (39.1 mg, 25%). ^1^H-NMR: (δ) 7.78 (1H, s, H-6), 5.17 (2H, s, H-1"), 4.62 (1H, t, OH, *J* = 5.4 Hz), 4.59 (1H, t, OH, *J* = 5.4 Hz), 3.45–3.56 (4H, m, H-3", H-4"), 3.84 (3H, s, OCH_3_), 3.62 (2H, t, H-2', *J* = 6.4 Hz), 2.45 (2H, t, H-1', *J* = 6.6 Hz) ppm. ^13^C-NMR: (δ) 171.7 (C-4), 155.8 (C-2), 146.8 (C-6), 105.1 (C-5), 78.3 (C-1"), 71.3 (C-3"), 60.5 (C-4"), 59.8 (C-2'), 54.7 (OCH_3_), 29.9 (C-1') ppm. MS (ESI): *m/z* = 245.1 ([*M*+H]^+^).

*5-(2-Hydroxyethyl)-N-1-[(1,3-dibenzyloxy-2-propoxy)methyl]pyrimidin-2,4-dione* (**26**) *and 5-(2-chloroethyl)-N-1-[(1,3-dibenzyloxy-2-propoxy)methyl]pyrimidin-2,4-dione* (**27**). *Method B*: A mixture of compound **1** (1.3 g, 8.33 mmol), hexamethyldisilazane (30 mL), trimethylsilyl chloride (0.58 mL, 4.57 mmol) and (NH_4_)_2_SO_4_ (416 mg, 3.15 mmol) was refluxed for 24 h under argon atmosphere. The resultant solution was evaporated in vacuum using oil pump. To the residual oil in acetonitrile (50 mL), TBAI (73.7 mg, 0.2 mmol) and 1,3-dibenzyloxy-2-chloromethoxypropane (4 g, 12.45 mmol) were added under argon atmosphere. The reaction mixture was stirred at reflux overnight. After the reaction was complete the solvent was evaporated and the residue purified using column chromatography (CH_2_Cl_2_–CH_3_OH = 35:1) to obtain compounds **26** (289.6 mg, 8%) and **27** (87.3 mg, 2%).

**26**: ^1^H-NMR: (δ) 11.30 (1H, s, NH), 7.53 (1H, s, H-6), 7.34–7.28 (10H, m, Ph), 5.21 (2H, s, H-1"), 4.56 (1H, t, OH, *J* = 5.4 Hz), 4.46 (4H, s, H-5"), 4.0−3.9 (1H, m, H-3") 3.50−3.40 (6H, m, H-2', H-4"), 2.30 (2H, t, H-1', *J* = 6.7 Hz) ppm. ^13^C-NMR: (δ) 164.4 (C-4), 151.6 (C-2), 142.1 (C-6), 138.7 (C-Ph), 128.7 (C-Ph), 127.8 (C-Ph), 127.8 (C-Ph), 111.0 (C-5), 77.1 (C-3"), 76.5 (C-1"), 72.7 (C-5"), 70.3 (C-4"), 60.0 (C-2'), 30.3 (C-1') ppm. MS (ESI): *m/z* = 441.3([*M*+H]^+^).

**27**: ^1^H-NMR: (δ) 11.43 (1H, s, NH), 7.69 (1H, s, H-6), 7.38–7.2 (10H, m, Ph), 5.20 (2H, s, H-1"), 4.46 (4H, s, H-5"), 4.04–3.95 (1H, m, H-3"), 3.62 (2H, t, H-2', *J* = 6.9 Hz), 3.55–3.40 (4H, m, H-4"), 2.58 (2H, t, H-1', *J* = 7.0 Hz) ppm. ^13^C-NMR: (δ) 164.1 (C-4), 151.5 (C-2), 143.0 (C-6), 138.7 (C-Ph), 128.7 (C-Ph), 127.8 (C-Ph), 127.7 (C-Ph), 109.8 (C-5), 77.2 (C-3"), 76.6 (C-1"), 72.7 (C-5"), 70.3 (C-4"), 43.5 (C-2'), 30.2 (C-1') ppm. MS (ESI): *m/z* = 459. 2 ([*M*+H]^+^).

*5-(2-Chloroethyl)-N-1-[(1,3-dihydroxy-2-propoxy)methyl]pyrimidin-2,4-dione* (**28**). *Method B*: In the similar way as described for compound **23**, the synthesis of **28** was performed. The reagents used were: compound **27** (43.7 mg, 0.1 mmol), anhydrous CH_2_Cl_2_ (8 mL) and BCl_3_ (0.7 mL, 0.7 mmol). After column chromatography (CH_2_Cl_2_–CH_3_OH = 5:1) compound **28** (15 mg, 57%) was isolated. ^1^H-NMR: (δ) 11.36 (1H, s, NH), 7.65 (1H, s, H-6), 5.13 (2H, s, H-1"), 4.58 (2H, t, 2×OH, *J* = 5.5 Hz), 3.66 (2H, t, H-2', *J* = 6.9 Hz), 3.51–3.32 (5H, m, H-3", H-4"), 2.62 (2H, t, H-1', *J* = 6.9 Hz) ppm. ^13^C-NMR: (δ) 164.2 (C-4), 151.3 (C-2), 143.2 (C-6), 110.7 (C-5), 81.0 (C-3"), 76.4 (C-1"), 61.4 (C-4"), 43.6 (C-2'), 30.3 (C-1') ppm. MS (ESI): *m/z* = 279.2([*M*+H]^+^).

*N-1-[4-Acetoxy-(3-acetoxymethyl)butyl]-5-(2-acetoxyethyl)-4-methoxypyrimidin-2-one* (**29**). To a stirred solution of compound **14** (76.3 mg, 0.36 mmol) and K_2_CO_3_ (74.5 mg, 0.54 mmol) in anhydrous DMF (7.6 mL), 4-acetoxy-(3-acetoxymethyl)butyl iodide (192 mg, 0.61 mmol) was added under argon atmosphere. The reaction mixture was stirred at 60 °C for 20 min and then evaporated to dryness. After column chromatography (CH_2_Cl_2_–CH_3_OH = 30:1) compound **29** was isolated as a colourless oil (87.1 mg, 60%). ^1^H-NMR: (δ) 7.89 (1H, s, H-6), 4.12 (2H, t, H-2', *J* = 6.6 Hz), 4.03 (4H, d, H-4", *J* = 5.8 Hz), 3.85 (3H, s, OCH_3_), 3.83 (2H, t, H-1", *J* = 7.7 Hz), 2.60 (2H, t, H-1', *J* = 6.6 Hz), 2.02 (6H, s, 2×COCH_3_), 1.99 (3H, s, COCH_3_), within COCH_3_ signal (H-3") 1.67 (2H, q, H-2", *J* = 7.1 Hz) ppm.^13^C-NMR: (δ) 170.8 (COCH_3_), 169.9 (C-4), 155.6 (C-2), 147.7 (C-6), 103.3 (C-5), 64.0 (C-4"), 62.6 (C-2'), 54.3 (OCH_3_), 47.2 (C-1"), 34.9 (C-3"), 27.9 (C-1') 26.0 (C-2"), 21.1 (COCH_3_) ppm. MS (ESI): *m/z* = 399.2 ([*M*+H]^+^).

*N-1-[4-Acetoxy-(3-acetoxymethyl)butyl]-5-(3-acetoxypropyl)-4-methoxypyrimidin-2-one* (**30**). The synthesis of **30** was performed as described for **29**. The reagents used were: compound **15** (1.54 g, 6.81 mmol), K_2_CO_3_ (1.41 g, 10.2 mmol), anhydrous DMF (40 mL) and 4-acetoxy-(3-acetoxymethyl)butyl iodide (3.63 g, 11.6 mmol). After column chromatography (CH_2_Cl_2_–CH_3_OH = 50:1) compound **30** was isolated as a yellow oil (1.15 g, 41%). ^1^H-NMR: (δ) 7.83 (1H, s, H-6), 4.04–3.98 (6H, m, H-4", H-3'), 3.84–3.80 (5H, m, OCH_3_, H-1"), 2.34 (2H, t, H-1', *J* = 7.5 Hz), 2.02 (6H, s, 2×COCH_3_), 2.00 (3H, s, COCH_3_), within COCH_3_ signal (H-3"), 1.78 (2H, k, H-2', *J* = 7.0 Hz), 1.67 (2H, q, H-2", *J* = 7.2 Hz) ppm. ^13^C-NMR: (δ) 170.9 (COCH_3_), 170.1 (C-4), 155.7 (C-2), 146.6 (C-6), 106.5 (C-5), 64.1 (C-4"), 63.6 (C-3'), 54.4 (OCH_3_), 47.2 (C-1"), 34.9 (C-3"), 27.9 (C-2'), 27.7 (C-2"), 22.9 (C-1'), 21.1 (COCH_3_), 21.1 (COCH_3_) ppm. MS (ESI): *m/z* = 413.2 ([*M*+H]^+^).

*5-(2-Hydroxyethyl)-N-1-[4-hydroxy-(3-hydroxymethyl)butyl]pyrimidin-2,4-dione* (**31**). The synthesis of **31** was performed as described for **19**. The reagents used were: compound **29** (62.4 mg, 0.16 mmol) and 1 M NaOH (5 mL). After column chromatography (CH_2_Cl_2_–CH_3_OH = 5:1) compound **31** was obtained as a white solid (28.2 mg, 70%, m.p.: 83–84 °C). ^1^H-NMR: (δ) 11.15 (1H, bs, NH), 7.46 (1H, s, H-6), 4.54 (1H, t, OH, *J* = 5.5 Hz), 4.40 (2H, t, 2×OH, *J* = 5.2 Hz), 3.69 (2H, t, H-1", *J* = 7.6 Hz), 3.45 (2H, q, H-2', *J* = 6.3 Hz), 3.42–3.32 (4H, m, H-4"), 2.32 (2H, t, H-1', *J* = 6.7 Hz), 1.55 (2H, q, H-2", *J* = 7.3 Hz), 1.47 (1 H, m, H-3", *J* = 5.9 Hz) ppm. ^13^C-NMR: (δ) 164.5 (C-4), 151.2 (C-2), 142.9 (C-6), 109.3 (C-5), 61.9 (C-4"), 59.9 (C-2'), 46.2 (C-1"), 41.2 (C-3"), 30.4 (C-1'), 28.2 (C-2") ppm. MS (ESI): *m/z* = 259.1 ([*M*+H]^+^). Anal. Calcd for C_11_H_18_N_2_O_5_: C, 51.15; H, 7.02; N, 10.85. Found: C, 51.10; H, 6.99; N, 10.89.

*5-(3-Hydroxypropyl)-N-1-[4-hydroxy-(3-hydroxymethyl)butyl]pyrimidin-2,4-dione* (**32**). The synthesis of **32** was performed in the same way as described for **31**. The reagents used were: compound **30** (204 mg, 0.49 mmol) and 1 M NaOH (5 mL). After column chromatography (CH_2_Cl_2_–CH_3_OH = 5:1) compound **32** was obtained as white crystals (110 mg, 82%, m.p.: 113–114 °C). ^1^H-NMR: (δ) 11.14 (1H, s, NH), 7.46 (1H, s, H-6), 4.43–4.38 (3H, m, OH), 3.70 (2H, t, H-1", *J* = 7.3 Hz), 3.46–3.30 (6H, m, H-4", H-3'), 2.22 (2H, t, H-1', *J* = 7.4 Hz), 1.63–1.42 (5H, H-2', H-2", H-3") ppm. ^13^C-NMR: (δ) 164.4 (C-4), 151.2 (C-2), 141.9 (C-6), 113.0 (C-5), 61.9 (C-4"), 60.6 (C-3'), 46.2 (C-1"), 41.2 (C-3"), 31.9 (C-2'), 28.3 (C-2"), 23.3 (C-1') ppm. MS (ESI): *m/z* = 273.2 ([*M*+H]^+^). Anal. Calcd for C_12_H_20_N_2_O_5_: C, 52.93; H, 7.40; N, 10.29. Found: C, 52.97; H, 7.43; N, 10.33.

### 3.3. Phosphorylation Assay of **19**, **21**, **23**, **31** and **32**

#### 3.3.1. Protein Expression

The HSV-1 TK was expressed in *E. coli* as a GST-TK fusion protein and further purified as HSV-1 TK-GST protein by using glutathione affinity chromatography following protocols that have been reported previously [33]. The human thymidine kinase 1 (hTK) was expressed in E. coli as a 6xHisTagged protein and purified following the previously published protocol [34].

#### 3.3.2. HPLC System and Conditions

The high liquid performance chromatography system used Merck HITACHI LaChrom (Gynkotek HPLC, Münich, Germany) equipped with a pump (L-7100), autosampler (L-7200) and a UV detector (L-7400). The analyses were performed on an LiChroCART® 250-4 cartridge column packed with a stationary phase made of spherical partical of silica (LiCrospher® 100 RP-18 endcapped, (5 µm)) and coupled with a guard column LiChroCART® 4-4 of the same type (Merck, KGaA, Darmstadt, Germany). Mobile phase consisted of 200 mM NaH_2_PO_4_, 25 mM tetrabutylammonium hydrogen sulfate (TBAHS) as the ion-pairing reagent and 0.5% methanol (HPLC grade). All aqueous solutions were prepared using de-ionized water from Millipore. The column was kept at a constant temperature of 20 °C. The separation was performed isocratically at a flow-rate of 1.1 mL/min and the UV detection was done at 254 nm [32].

#### 3.3.3. The Phosphorylation Reaction

Phosphorylation of dT and compounds **19**, **21**, **23**, **31**, fluorinated derivative of **31** and **32** was monitored by HPLC. Reactions were carried out in a final volume of 70 µL containing 50 mM HEPES pH 7.5, 5 mM ATP, 5 mM MgCl_2_, 1 mM solution of dT and tested compounds and either 11 µg of purified HSV-1 TK-GST or 0.8 µg of hTK. As the solutions of dT and tested compounds were prepared with 100% DMSO attention was paid for not adding more than 5% DMSO in the sample reaction (the final concentration of DMSO was 1%). The reaction was started with the addition of HSV-1 TK-GST. The reaction was done at 37 °C and was stopped after 30, 60 and 90 min by a 10-fold dilution with 50 mM EDTA (final concentration 5 mM) used to chelate the cofactor Mg^2+^. Each reaction was done in triplicate. Finally, a volume of 40 µL of the sample reaction was injected for analysis. The formation of the nucleotide monophosphate was monitored qualitatively. Two different blank reactions (no enzyme or no compound) were performed under the same experimental conditions to account for the background ATP hydrolysis. Control reactions were stopped after 30, 60 and 90 min (Supplementary Materials). The resulting ADP/ATP ratios were calculated and used as a measure for protein activity. The mean of at least triplicate measurements are reported.

#### 3.3.4. Cellular Activity Evaluations

The WI38 (normal diploid human fibroblasts) cell lines were cultured as monolayers and maintained in Dulbecco’s modified Eagle medium (DMEM) supplemented with 10% fetal bovine serum (FBS), 2 mM L-glutamine, 100 U/mL penicillin and 100 μg/mL streptomycin in a humidified atmosphere with 5% CO_2_ at 37 °C. The panel cell lines were inoculated onto a series of standard 96-well microtiter plates on day 0, at 3000 cells to 6000 cells per well according to the doubling times of specific cell line. Test agents were then added in five, 10-fold dilutions (1 × 10^−^^8^ to 1 × 10^−^^4^ M) and incubated for further 72 h. Working dilutions were freshly prepared on the day of testing in the growth medium. The solvent (DMSO) was also tested for eventual inhibitory activity by adjusting its concentration to be the same as in the working concentrations (DMSO concentration never exceeded 0.1%). After 72 h of incubation, the cell growth rate was evaluated by performing the MTT assay: experimentally determined absorbance values were transformed into a cell percentage growth (PG) using the formulas proposed by NIH and described previously [35].This method directly relies on control cells behaving normally at the day of assay because it compares the growth of treated cells with the growth of untreated cells in control wells on the same plate—the results are therefore a percentile difference from the calculated expected value. The IC_50_ and LC_50_ values for each compound were calculated from dose-response curves using linear regression analysis by fitting the mean test concentrations that give PG values above and below the reference value. If, however, all of the tested concentrations produce PGs exceeding the respective reference level of effect (e.g., PG value of 50) for a given cell line, the highest tested concentration is assigned as the default value (in the screening data report that default value is preceded by a “>” sign). Each test point was performed in quadruplicate in three individual experiments. The results were statistically analyzed (ANOVA, Tukey post-hoc test at *p* < 0.05). Finally, the effects of the tested substances were evaluated by plotting the mean percentage growth for each cell type in comparison to control on dose-response graphs.

## 4. Conclusions

5-(2-Hydroxyethyl)- and 5-(3-hydroxypropyl)-substituted 4-methoxypyrimidin-2-ones (**10** and **14**–**16**) were prepared by an efficient synthetic route and subjected to N-1 alkylation to obtain target compounds bearing 2,3-dihydroxypropyl (**19**), acyclovir- (**21**), ganciclovir- (**23**), and penciclovir-like side chains (compounds **31**, **32**). The two synthetic methods A and B that were applied for *N*-alkylation indicated that method A using activated *N*-anionic 5-substituted pyrimidine derivatives was more successful than the silylation reaction method.

The influence of the acyclic glycone in target compounds **19**, **21**, **23**, **31** and **32** on their phosphorylation ability was observed. Thereby, 5-substituted pyrimidine derivatives **31** and **32** bearing N-1 penciclovir-like side chain revealed to be substrates of HSV-1 TK. The structural congener of **31** bearing an oxygen isosteric replacement of methylene group in side chain of **23** was not phosphorylated by HSV-1 TK. Moreover, 5-(2-hydroxyethyl)pyrimidin-2,4-dione **31** showed to be a better substrate when compared to compound **32** with a longer substituent bearing an additional methylene group. 

Besides, both compounds **31** and **32** exhibited no cytotoxic effect against normal human fibroblasts up to 100 µM. Compound **31** has been selected as a lead compound for development as a new PET probe for imaging HSV-1 TK expression. Therefore, the fluorine-18 radiolabeling of the corresponding pyrimidine precursor of **31** and the subsequent *in vivo* PET studies are currently underway.

## Supplementary Materials

Supplementary Materials containing HPLC chromatograms of phosphorylation reaction of compounds **19**, **21** and **23** with HSV-1 TK and hTK, and HPLC chromatograms for time conversion (30, 60 and 90 min of incubation) in phosphorylation of dT, **31** and its fluorinated derivative, as well as blank reactions (no enzyme or no substrate) can be accessed at: http://www.mdpi.com/1420-3049/18/5/5104/s1.
