# Supplementary material for: C-5 Hydroxyethyl and Hydroxypropyl Acyclonucleosides as Substrates for Thymidine Kinase of Herpes Simplex Virus Type 1 (HSV-1 TK): Syntheses and Biological Evaluation
Source: Molecules. 2013 May 2;18(5):5104–24. doi: 10.3390/molecules18055104 (PMC6270122; doi:10.3390/molecules18055104)
Supplement: Supplementary file 1 [file molecules-18-05104-s001.pdf]

Supplementary Materials

1. Phosphorylation Assay of Compound 19

Figure S1. HPLC chromatograms of the reaction mixture of compound **19**, the enzyme (HSV-1 TK or hTK) and ATP.

HSV-1 TK hTK

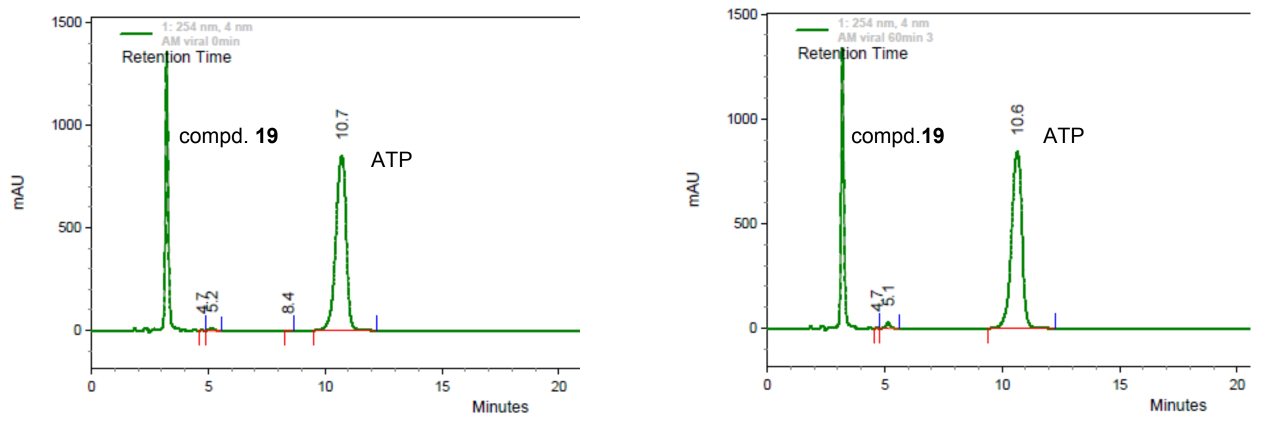

Formation of a new peak corresponding to monophosphated derivative of **19** was not detected. Compound **19** is not a substrate for HSV-1 TK and human tymidine kinase (hTK).

2. Phosphorylation Assay of Compound 21

Figure S2. HPLC chromatograms of the reaction mixture of compound **21**, the enzyme (HSV-1 TK or hTK) and ATP.

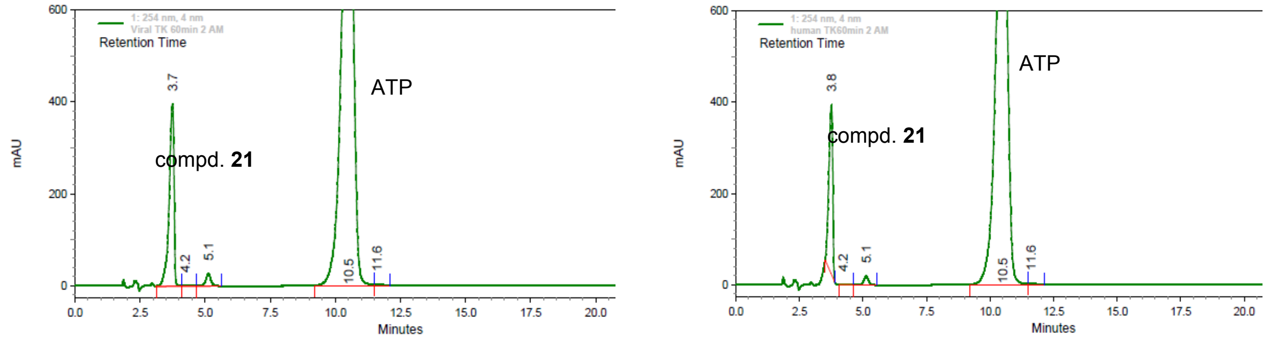

Formation of a new peak corresponding to monophosphated derivative of **21** was not detected. Compound **21** is not a substrate for HSV-1 TK and hTK.

3. Phosphorylation Assay of Compound 23

Figure S3. HPLC chromatograms of the reaction mixture of compound **23**, the enzyme (HSV-1 TK or hTK) and ATP.

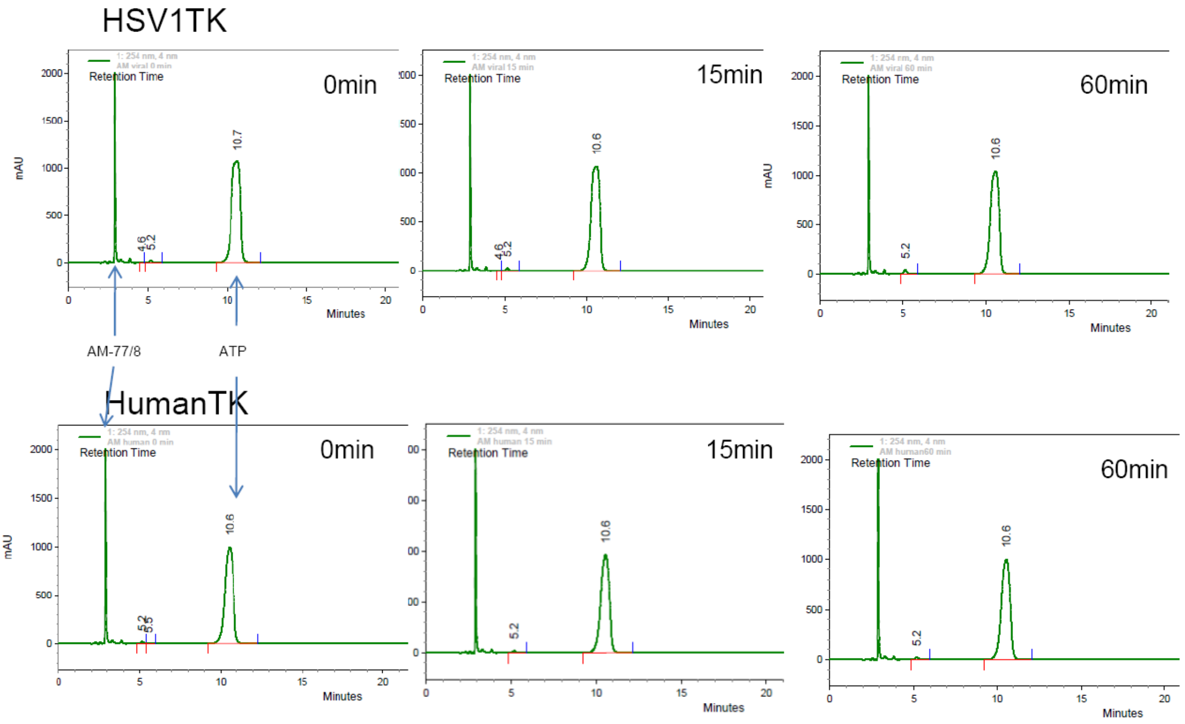

Formation of a new peak which would correspond to monophosphated derivative of **23** was not detected suggesting that **23** is not a substrate for HSV1 TK and hTK.

4. Phosphorylation Assays of dT and Compound 31

Figure S4. HPLC chromatograms of dT (1 mM) during incubation with HSV-1 TK and ATP, and blank reactions (no enzyme or no dT). Reactions were stopped after (**a**) 30 min, (**b**) 60 min, (**c**) 90 min.

(**a**) at 30 min

Blank 1 (without HSV-1 TK) Blank 2 (without dT)

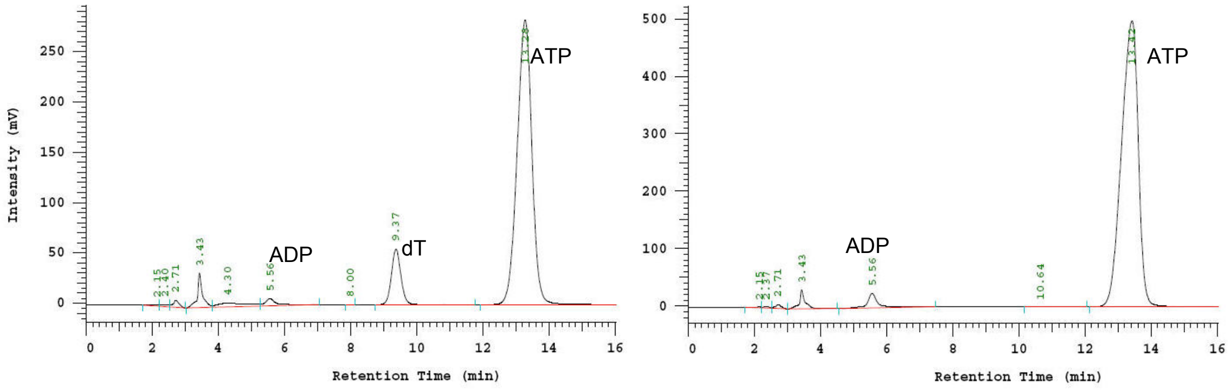

Reaction at 30 min

Figure S4. *Cont.*

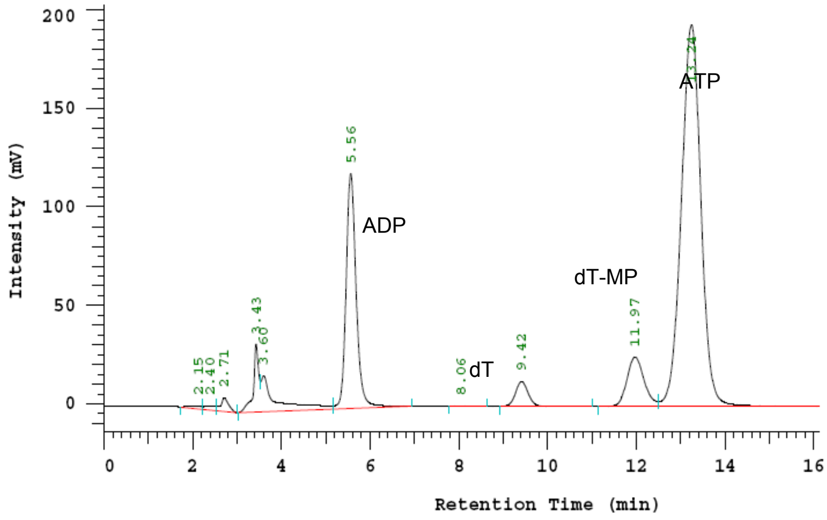

(**b**) at 60 min

Blank 1 (without HSV-1 TK)

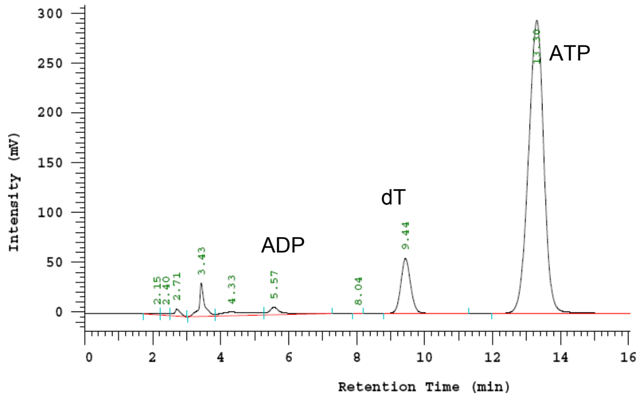

Blank 2 (without dT)

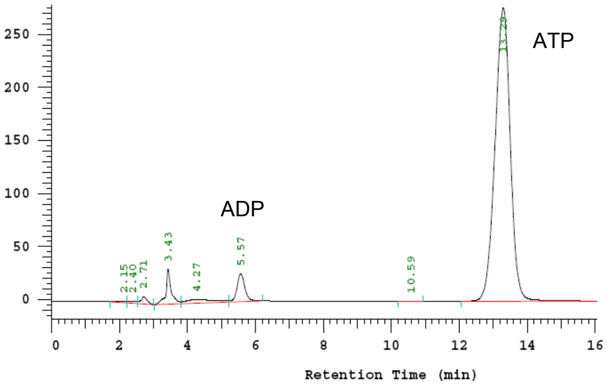

Reaction at 60 min

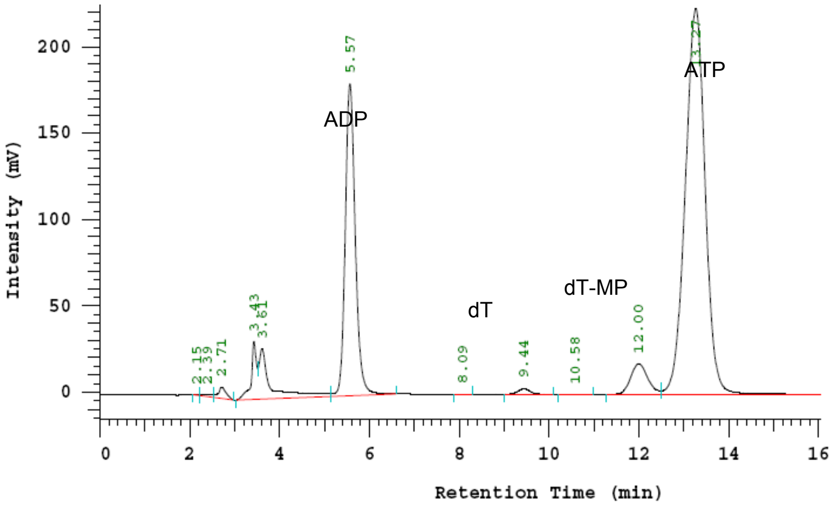

Figure S4. *Cont.*

(**c**) at 90 min

Blank 1 (without HSV-1 TK)

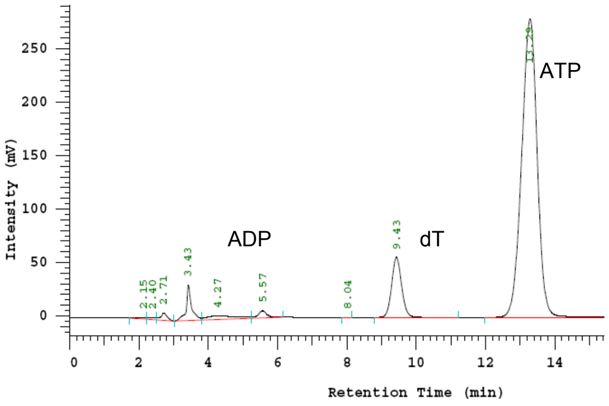

Blank 2 (without dT)

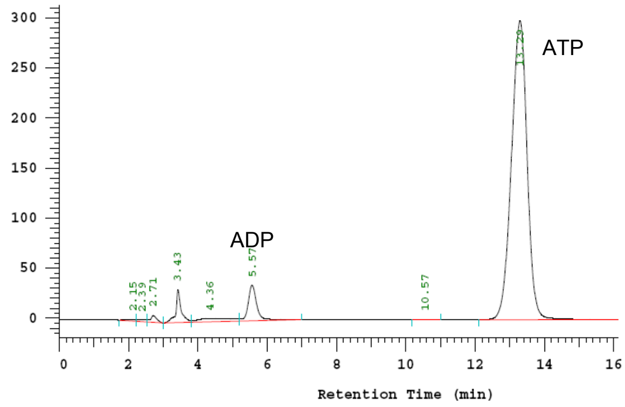

Reaction at 90 min

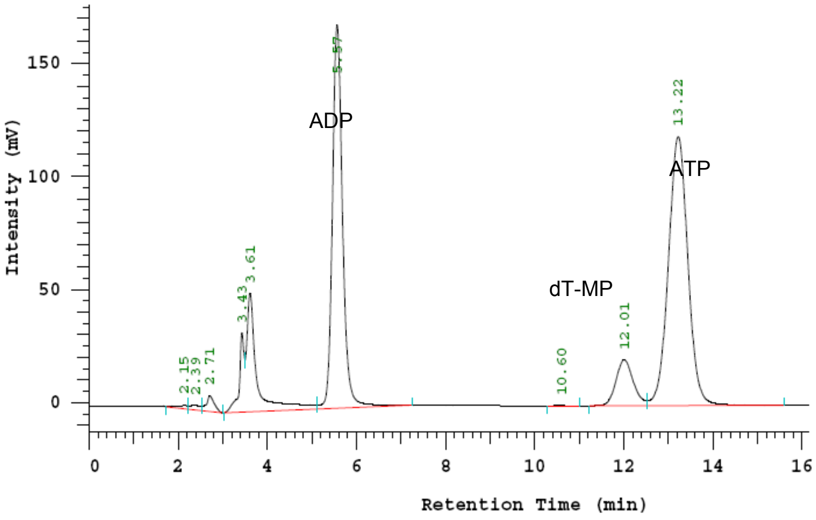

Figure S5. HPLC chromatograms of the reaction mixture of compound **31**, HSV-1 TK and ATP, and blank reactions (no enzyme or no compound **31**). Reactions were stopped at (**a**) 30 min, (**b**) 60 min, (**c**) 90 min. Elution with mobile phase 0.2 M NaH_2PO_4, 25 mM tetrabutylammonium hydrogen sulfate and 1 % methanol at flow rate 1 mL/min and column LiCrospher® 100 RP-18 endcapped, (5 µm) did not allow an efficient separation of compound **31** and ADP. Blank reactions were run concomitantly with reaction experiments to account for background ATP hydrolysis.

(**a**) at 30 min

Blank 1 (without HSV-1 TK)

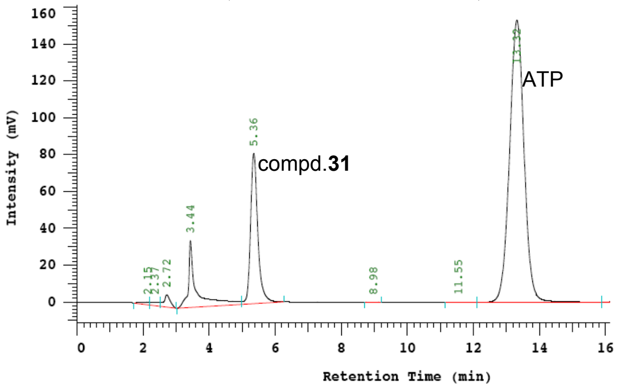

Blank 2 (without compound **31**)

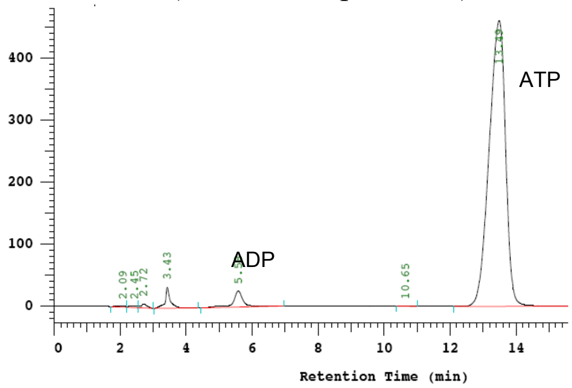

Reaction at 30 min

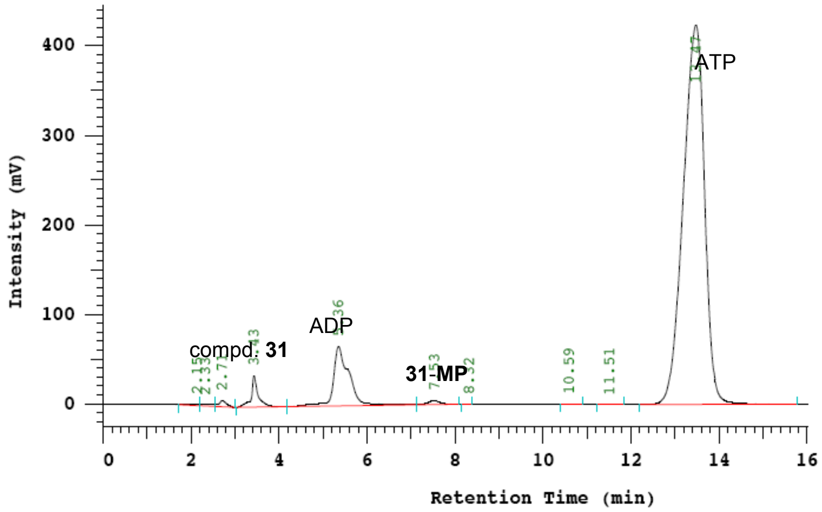

(**b**) at 60 min

Blank 1 (without HSV-1 TK)

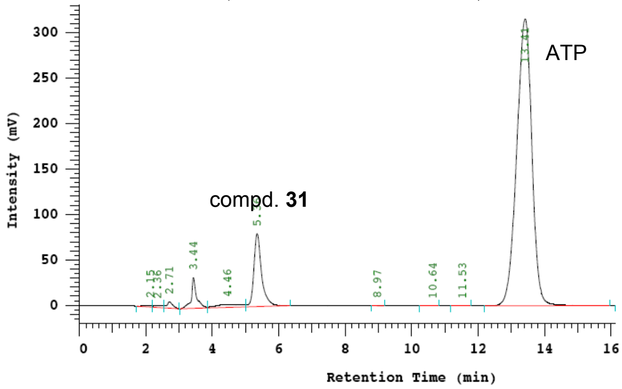

Blank 2 (without compound **31**)

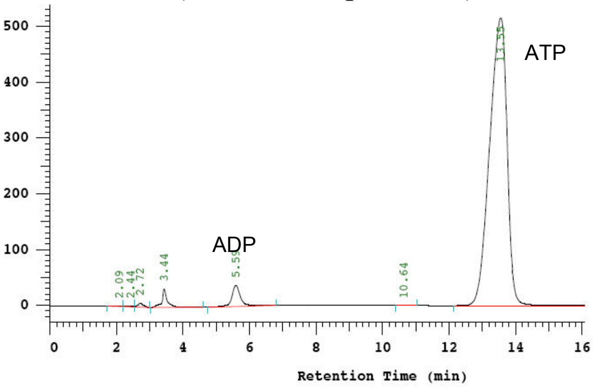

Figure S5. *Cont.*

Reaction at 60 min

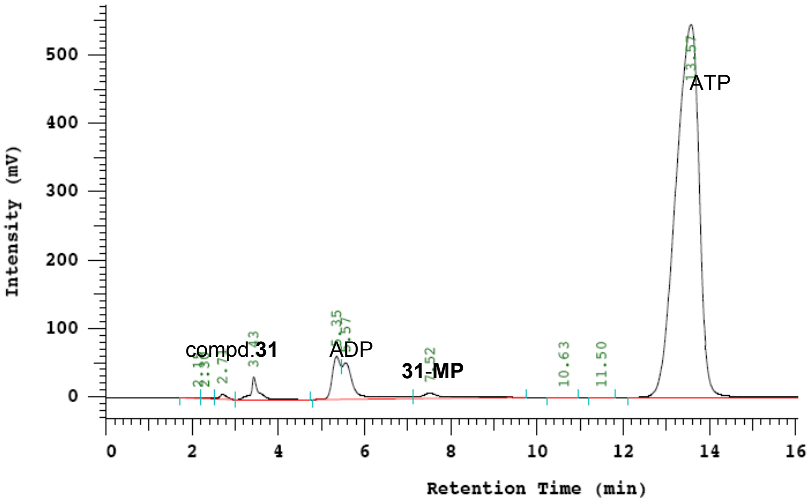

(**c**) at 90 min

Blank 1 (without HSV-1 TK)

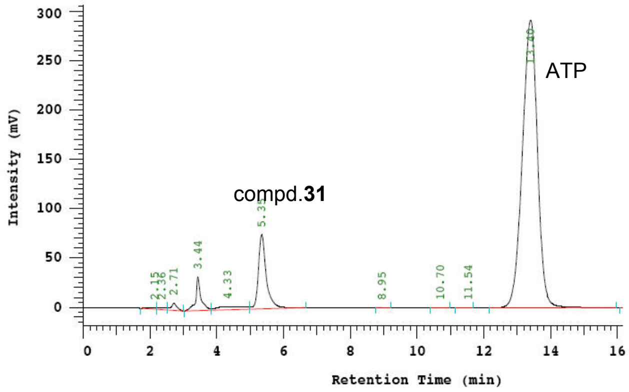

Blank 2 (without compound **31**)

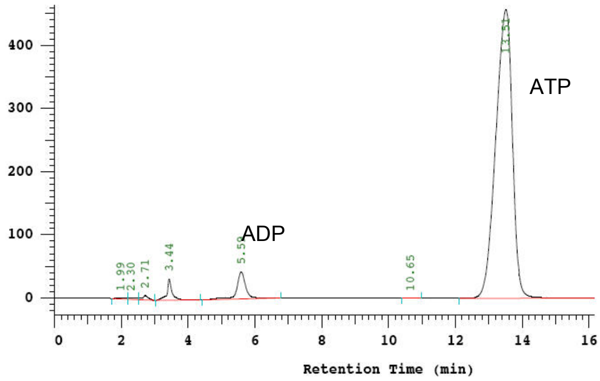

Reaction at 90 min

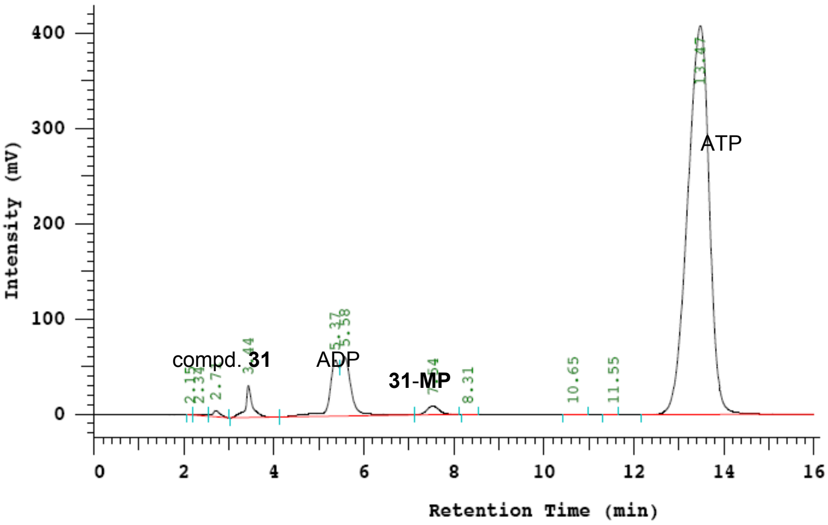

Formation of new peak corresponding to the monophosphated derivative of **31** (**31-MP**) is observed. Increase of peaks that can be ascribed to both **31-MP** and ADP can be seen during time, as well as decrease of the peak corresponding to compound **31**.

5. Phosphorylation Assay of Fluorinated Derivative of 31 (F-31)

Figure S6. HPLC chromatograms of the reaction mixture of **F-31**, HSV-1 TK and ATP, and blank reactions (no enzyme or no **F-31**). Reactions were stopped at (**a**) 30 min, (**b**) 60 min, (**c**) 90 min.

(**a**) at 30 min

Blank 1 (without HSV-1 TK)

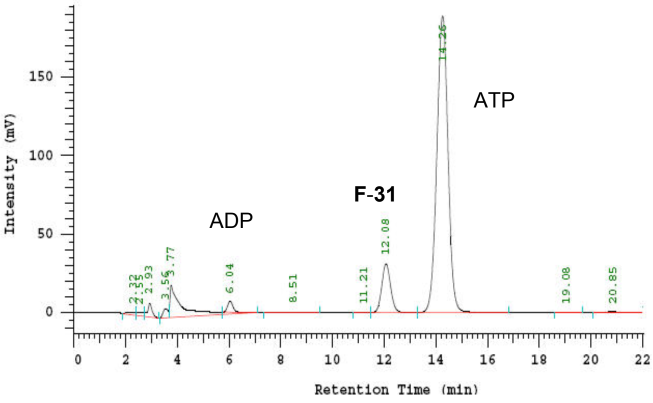

Blank 2 (without **F-31**)

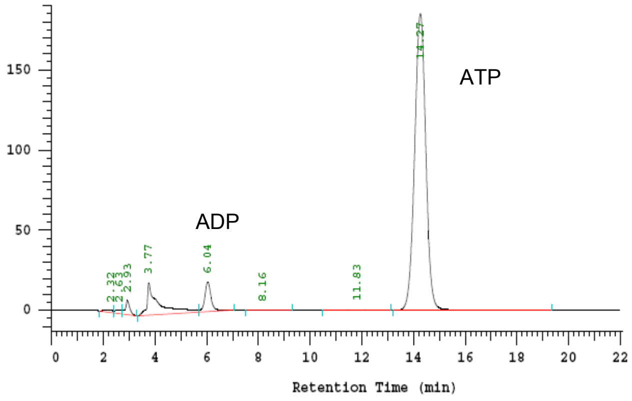

Reaction at 30 min

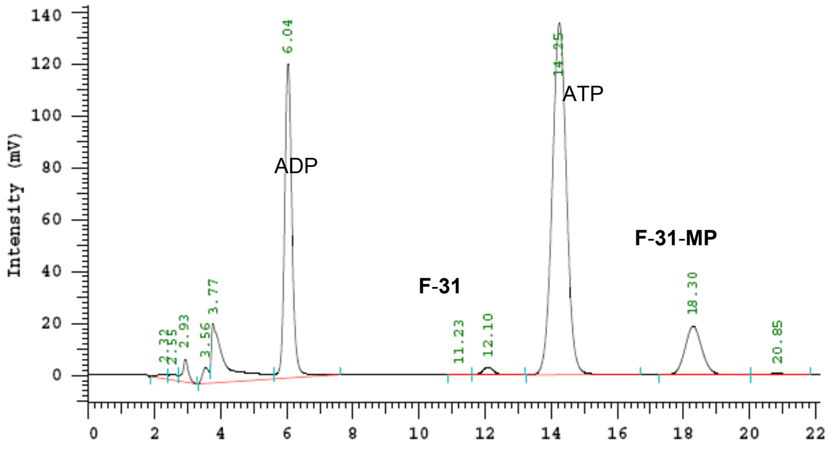

(**b**) at 60 min

Blank 1 (without HSV-1 TK)

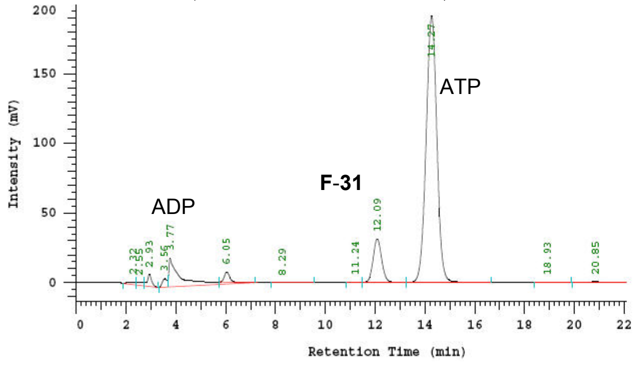

Blank 2 (without **F-31**)

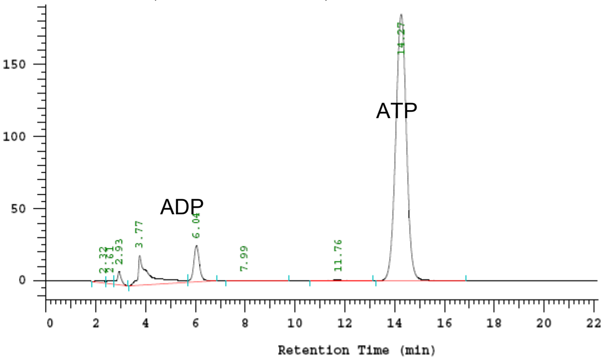

Reaction at 60 min

Figure S6. *Cont.*

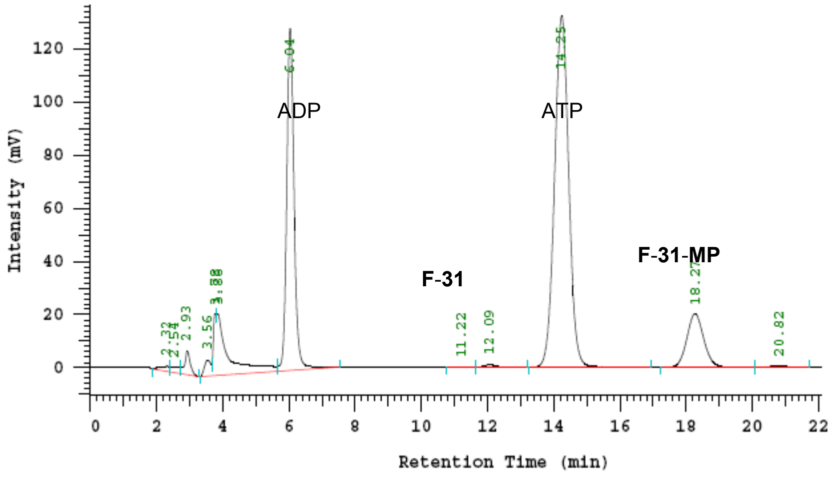

(**c**) at 90 min

Blank 1 (without HSV-1 TK)

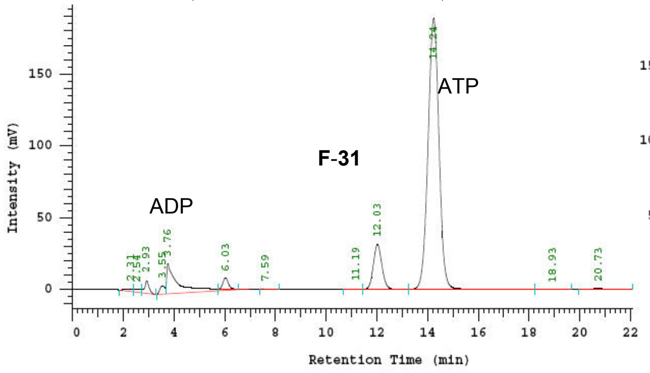

Blank 2 (without **F-31**)

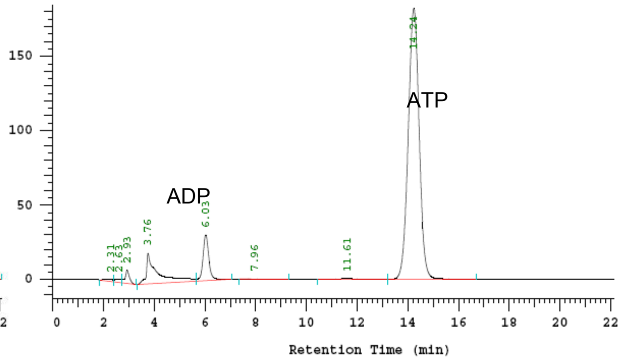

Reaction at 90 min

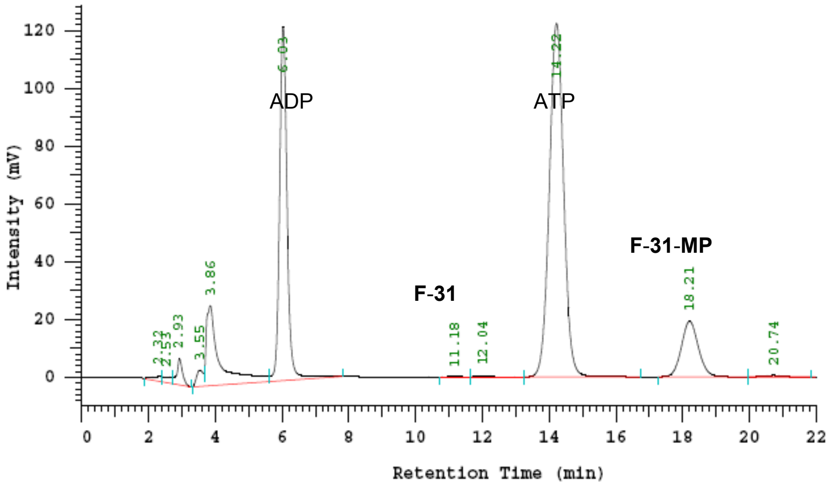

Formation of new peak corresponding to the monophosphated derivative of **F-31** (**F-31-MP)** is observed. Increase of peaks that can be ascribed to both **F-31-MP** and ADP can be observed during incubation, as well as decrease of the peak corresponding to **F-31**. Compound **F-31** is almost entirely converted to monophosphate **F-31-MP** during 90 min of incubation.
